# Rifaximin Prevents T-Lymphocytes and Macrophages Infiltration in Cerebellum and Restores Motor Incoordination in Rats with Mild Liver Damage

**DOI:** 10.3390/biomedicines9081002

**Published:** 2021-08-12

**Authors:** Tiziano Balzano, Paola Leone, Gergana Ivaylova, M. Carmen Castro, Lestteriel Reyes, Chusé Ramón, Michele Malaguarnera, Marta Llansola, Vicente Felipo

**Affiliations:** Laboratory of Neurobiology, Centro Investigación Príncipe Felipe, 46012 Valencia, Spain; tbalzano.hmcinac@hmhospitales.com (T.B.); paolairmaleone@gmail.com (P.L.); givaylova@cipf.es (G.I.); mcastro@cipf.es (M.C.C.); lestteriel.reyes@gmail.com (L.R.); cramonvalero@gmail.com (C.R.); michele.malaguarnera@gmail.com (M.M.); vfelipo@cipf.es (V.F.)

**Keywords:** liver disease, minimal hepatic encephalopathy, chemokines, neuroinflammation, neurotransmission, cerebellum

## Abstract

In patients with liver cirrhosis, minimal hepatic encephalopathy (MHE) is triggered by a shift in peripheral inflammation, promoting lymphocyte infiltration into the brain. Rifaximin improves neurological function in MHE by normalizing peripheral inflammation. Patients who died with steatohepatitis showed T-lymphocyte infiltration and neuroinflammation in the cerebellum, suggesting that MHE may already occur in these patients. The aims of this work were to assess, in a rat model of mild liver damage similar to steatohepatitis, whether: (1) the rats show impaired motor coordination in the early phases of liver damage; (2) this is associated with changes in the immune system and infiltration of immune cells into the brain; and (3) rifaximin improves motor incoordination, associated with improved peripheral inflammation, reduced infiltration of immune cells and neuroinflammation in the cerebellum, and restoration of the alterations in neurotransmission. Liver damage was induced by carbon tetrachloride (CCl_4_) injection over four weeks. Peripheral inflammation, immune cell infiltration, neuroinflammation, and neurotransmission in the cerebellum and motor coordination were assessed. Mild liver damage induces neuroinflammation and altered neurotransmission in the cerebellum and motor incoordination. These alterations are associated with increased TNFa, CCL20, and CX3CL1 in plasma and cerebellum, IL-17 and IL-15 in plasma, and CCL2 in cerebellum. This promotes T-lymphocyte and macrophage infiltration in the cerebellum. Early treatment with rifaximin prevents the shift in peripheral inflammation, immune cell infiltration, neuroinflammation, and motor incoordination. This report provides new clues regarding the mechanisms of the beneficial effects of rifaximin, suggesting that early rifaximin treatment could prevent neurological impairment in patients with steatohepatitis.

## 1. Introduction

Patients with liver cirrhosis may show minimal hepatic encephalopathy (MHE), with mild cognitive impairment, psychomotor slowing, attention deficits, and motor incoordination. MHE reduces the ability to perform daily tasks, quality of life, and life span and increases accidents, falls, and hospitalizations. MHE is a serious health, social, and economic problem [1]. The mechanisms by which liver disease induces MHE are beginning to be clarified in studies in patients and animal models.

Cognitive and motor alterations have been prevented or reversed in rats with hyperammonemia and MHE by treatments that reduce peripheral inflammation, such as anti-TNF-a or ibuprofen [2,3,4,5,6], and microglia activation and neuroinflammation by treatments such as p38 inhibitors, sulforaphane, phosphodiesterase 5 inhibitors, or extracellular cGMP [7,8,9,10], or treatments that reduce GABAergic neurotransmission [11]. These studies support the idea that liver damage and hyperammonemia lead to peripheral inflammation, which induces neuroinflammation, which alters neurotransmission, resulting in impaired cognitive and motor function (reviewed in [12]).

A similar process would be expected to occur in patients with chronic liver disease. Mangas-Losada et al. [13] proposed that the appearance of MHE in patients with liver cirrhosis is associated with a shift in the type of peripheral inflammation, resulting in increased activation and differentiation of CD4^+^ T lymphocytes to Th17, Th follicular, and Th22; an increased amount of CD4^+^CD28^−^T lymphocytes; and increased IL-17, CCL20, CX3CL1 (fractalkine), and IL-15 levels. They proposed that this shift in peripheral inflammation would promote the infiltration of lymphocytes into the brain [13], which is promoted by CCL20, CX3CL1, and IL-15 [14,15,16]. This would induce neuroinflammation, resulting in cognitive and motor alterations in patients with MHE.

Patients with mild steatohepatitis show infiltration of T lymphocytes in the meningeal space of the cerebellum, associated with neuroinflammation, microglia and astrocyte activation, and loss of Purkinje and granular neurons [17]. Moreover, patients with steatohepatitis show mild cognitive and motor impairment before reaching liver cirrhosis if ammonia and inflammation levels are high enough [18]. This supports the idea [13] that a shift in peripheral inflammation leading to lymphocyte infiltration into the brain would trigger neuroinflammation and MHE in cirrhotic patients [13]. Moreover, this process would already occur in patients with mild steatohepatitis, before reaching cirrhosis [17].

T-lymphocytes infiltration, neuroinflammation, and neuronal loss are remarkable in the cerebellum [17], the function of which is altered in early MHE phases [19], as reflected in early alterations of motor coordination modulated by the cerebellum [20,21]. These reports suggest that changes in the immune system and T-lymphocyte infiltration into the brain associated with the appearance of mild cognitive and motor impairment may already occur in the early phases of steatohepatitis. If this is the case, treatments to prevent or reverse this process and MHE should be used in patients with steatohepatitis, before reaching liver cirrhosis.

Treatment with rifaximin, a nonpermeable antibiotic, improves cognitive and motor function in a relevant proportion of patients with MHE [22,23], and this is associated with normalization of peripheral IL-17, CCL20, CX3CL1, and IL-15 [23], which promote the infiltration of lymphocytes into the brain. This, together with the above reports, suggests that rifaximin could improve MHE by reducing T lymphocytes infiltration into the brain. This cannot be assessed in patients but can be studied in animal models. It should also be kept in mind that eventual beneficial effects of rifaximin in brain alterations will be indirect and mediated through a reduction of peripheral inflammation, since this molecule is not absorbed in the intestine.

The aims of this work were to assess in a rat model of mild liver damage whether: (1) the rats show impaired motor coordination in the early phases of liver damage; (2) this is associated with changes in the immune system and infiltration of immune cells into the brain; and (3) rifaximin improves motor incoordination, associated with improved peripheral inflammation, reduced infiltration of immune cells and neuroinflammation in the cerebellum, and restoration of the alterations in neurotransmission.

As a rat model of chronic liver disease, we used rats injected with carbon tetrachloride (CCl_4_). CCl_4_ creates aspects of pathology similar to those observed in patients with steatohepatitis and liver damage, which progress from steatosis to different grades of liver inflammation, fibrosis, and liver cirrhosis [24,25].

## 2. Materials and Methods

### 2.1. Animal Model and Treatment with Rifaximin 

Male Wistar rats (150–180 g) were intraperitoneally injected (1 mL/kg) 3 times/week over four weeks with 10% CCl_4_ dissolved in corn oil to induce mild liver damage [26]. Control rats were injected with corn oil. Rats were distributed into four groups: control, control+rifaximin, CCl_4_, and CCl_4_+rifaximin. Rifaximin (Sigma) was dissolved in 100% ethanol (20 mg/kg) and orally administered (20–50 μL of ethanol according to body weight) once daily until sacrifice. Control rats received the corresponding volume of 100% ethanol.

The experiments were approved by the Comite Ético de Experimentación Animal (CEEA) of our center and by the Conselleria de Agricultura of Generalitat Valenciana and performed according to the European Commission Directive (2010/63/EU) for the care and management of experimental animals and complied with the ARRIVE guidelines for animal research. The number of rats used for each parameter is indicated in the corresponding figure caption.

### 2.2. Experimental Design 

**Figure 1:**
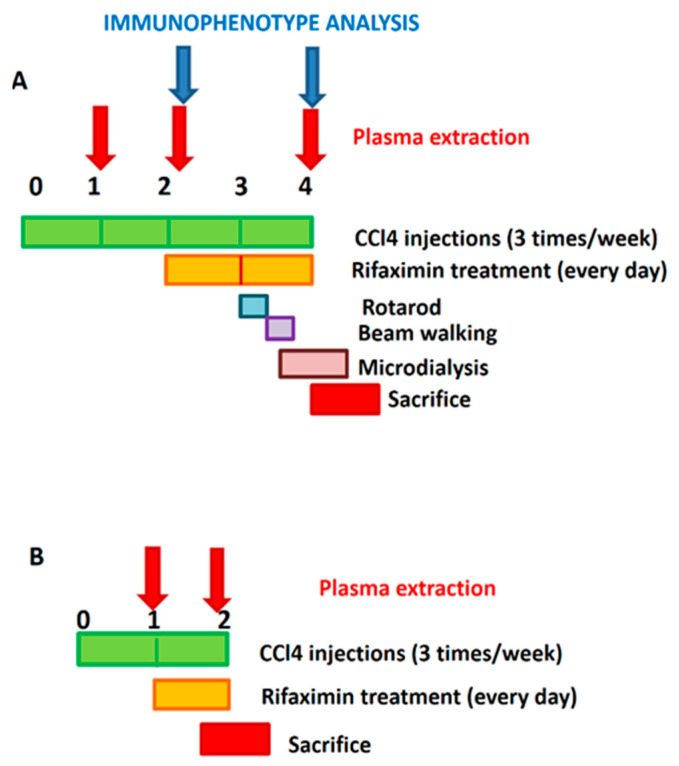
Experimental design. (**A**) In most experiments, treatment with rifaximin started 2 weeks after the first CCl_4_ injection and was maintained daily until sacrifice, at the end of the fourth week. Blood from saphenous vein was taken at 1, 2, and 4 weeks for analysis of plasma cytokines and at 2 and 4 weeks for immunophenotype analysis. Motor coordination was assessed at the beginning of the fourth week. In vivo microdialysis was performed at 4 weeks. (**B**) To analyze effects of rifaximin on initial alterations, some experiments were carried out starting treatment 1 week after the first administration of CCl_4_. Daily treatment with rifaximin started at the beginning of the second week and maintained during the entire week. Rats were sacrificed at the end of the second week. Blood was taken for analysis of plasma cytokines at the beginning of the first and end of the second week.

The experimental design is summarized in Figure 1.

A. Rifaximin started 2 weeks after the first CCl_4_ injection and was maintained daily until sacrifice, at the end of the fourth week. Blood from the saphenous vein was taken at 1, 2, and 4 weeks for analysis of plasma cytokines and at 2 and 4 weeks for immunophenotype analysis. Motor coordination was assessed at the beginning of the fourth week. In vivo microdialysis was performed at 4 weeks.

B. To analyze the effects of rifaximin on the initial alterations, some experiments were carried out starting treatment with rifaximin one week after the first administration of CCl_4_. Daily treatment with rifaximin started at the beginning of the second week and maintained during the entire week. Rats were sacrificed at the end of the second week. Blood was taken for the analysis of plasma cytokines at the beginning of the first and end of the second weeks.

### 2.3. Liver Histology 

The grade of liver damage was analyzed using hematoxylin and eosin (H&E) and Masson trichrome stains of PFA-fixed paraffin-embedded livers. Liver damage was graded using a scoring system as in [27]. The histological features taken into account were steatosis (0–3), lobular inflammation (0–3), and fibrosis grade (0–3).

Analysis of cytokines in plasma: Plasma was obtained in vials containing EDTA and stored at −80 °C. TNF-a in plasma was measured using an ELISA kit from eBioscience (Waltham, MA, USA). All other cytokines in plasma were analyzed by Western blot. Primary antibodies used were against IL-17 (ab79056), IL-4 (ab9811), IL-10 (ab9969), and CCl20 (ab9829) 1:1000 from Abcam; TGF-β (PA5-99186) 1:1000, CX3CL1 (14-7986) 1:1000, and CCl5 (710001) 1:500 from Invitrogen (Waltham, MA, USA); IL-6 (ARC0062) 1:500 from BioSource (San Diego, CA, USA); IL-15 (1829R) 1:200 from BIOSS (Woburn, MA, USA); IFN-γ (MAB5851) 1:1000 from R&D Systems (Minneapolis, MN, USA); and CCl2 (66272) 1:1000 from Proteintech (Rosemont, IL, USA). Secondary antibodies (1:4000) were IgGs conjugated with alkaline phosphatase (Sigma, Madrid, Spain). The images were captured using a Hewlett Packard ScanJet 5300C and band intensities were quantified using AlphaImager 2200 software.

### 2.4. Characterization of Lymphocyte Population in Whole Blood by Flow Cytometry

Blood samples were taken in BD Microtainer^®^ tubes with EDTA. Whole blood was incubated with RBC Lysis Buffer Multi-species (eBioscience™) (2 mL per each 100 µL of blood) for 10 min at room temperature in the dark. After 2 washes with staining buffer (phosphate-buffered saline (PBS) with 2 mM EDTA and 0.5 % bovine serum albumin (BSA)), cells were incubated with Fc block (Purified Mouse Anti-Rat CD32, 1:100, BD Bioscience, Madrid, Spain) for 20 min on ice. Cells were harvested and incubated with a mixture of monoclonal antibodies specific for surface markers of different lymphocyte subpopulations for 30 min in the dark. After 2 washes with staining buffer, a Foxp3/Transcription Factor Staining Buffer Set (eBioscience) was used, and cells were stained for FoxP3. Two more washes were performed, and a viability marker (Zombie Yellow Fixable Viability Kit, 1:100; BioLegend, San Diego, CA, USA) was added. Antibodies used were: anti-CD45 PEVio770 (clone REA504, 1:20) for total lymphocytes, anti-CD3 FITC (clone REA223, 1:100) for T lymphocytes, anti-CD4 VioBlue (clone REA482, 1:100) for T helper (Th) lymphocytes, and anti-CD8a PercPVio700 (clone REA437, 1:10) for T cytotoxic lymphocytes, all from Miltenyi (Bergisch Gladbach, Germany); anti-CD28 BV786 (clone JJ319, 1:10) for negative selection of autoreactive Th lymphocytes and anti-CD25 BV786 (clone M-A251, 1:50) from BD Bioscience with anti-FoxP3 PE (clone FJK-16s, 1:25) from BioLegend for regulatory T lymphocytes. Samples were analyzed with a CytoFLEX cytometer and CytExpert software (Beckman Coulter, Brea, CA, USA).

### 2.5. Immunohistochemistry, Immunofluorescence, and Histological Staining of Cerebellar Sections 

Rats were anaesthetized with sodium pentobarbital and transcardially perfused with 0.9% saline followed by 4% paraformaldehyde in 0.1 M phosphate buffer (pH 7.4). Brains were removed and post-fixed in the same fixative solution for 24 h at 4 °C. Five-micrometer thick, paraffin-embedded sections were cut and mounted on coated slides.

#### 2.5.1. Immunohistochemistry Staining of Cerebellar Sections

The tissue sections were processed with an Envision Flex+kit (DAKO), blocking endogenous peroxidase activity for 5 min, and then incubated with primary antibodies. The reaction was visualized by Envision Flex + horseradish peroxidase for 20 min and then with diaminobenzidine for 10 min. Sections were counterstained with Mayer’s hematoxylin for 5 min. Primary antibodies used for the study were: anti-CD4 (Dako, Santa Clara, CA, USA, ready to use, or Abcam (Cambridge, UK, ab846), 1:50 for 20 min), anti-IBA1 (Wako, Osaka, Japan, 1:300 for 30 min), anti-GFAP (Dako, ready to use, for 20 min), anti-TNFa (ab6671 from Abcam, Cambridge, UK, 1:2000 for 45 min), anti-CCl2 (66272, Proteintech, Rosemont, IL, USA, 1:1000), anti-CCl20 (ab9829, Abcam; 1:200 overnight), anti-CX3CL1 (14-7986 from Invitrogen, Waltham, MA, USA, 1:200 overnight).

#### 2.5.2. Immunofluorescence in Cerebellar Sections

Double immunofluorescence was performed to analyze the subtypes of infiltrating lymphocytes using the following antibodies: total Th lymphocytes were marked with anti-CD4 (ab846 from Abcam, mouse 1:50 overnight at 4 °C; or NBP1-19371 from Novus (Centennial, CO, USA), rabbit 1:20 overnight at 4 °C). Anti-CX3CR1 (ab8021 from Abcam, rabbit 1:50 overnight at 4 °C) was used as marker of autoreactive CD28- lymphocytes and anti-CCR6 (MAB195 from R&D Systems (Minneapolis, MN, USA), mouse 1:100 overnight at 4 °C) as marker of Th17 lymphocytes. These were followed by Alexa 488 donkey anti-mouse and Alexa 647 donkey anti-rabbit (both 1:400, 1 h at room temperature) secondary antibodies, and for Th17 lymphocytes, Alexa 488 goat anti-rabbit and Alexa 555 goat anti-mouse (1:400, 1 h at room temperature) secondary antibodies were used. Nuclei were stained with DAPI (1:10,000) for 5 min. Staining was visualized with a Leica confocal microscope.

#### 2.5.3. Histological Staining of Cerebellar Sections

For histological analysis of cerebellum, H&E stain was performed.

### 2.6. Determination of Protein Content in Cerebellum by Western Blot 

Primary antibodies used were against CCl2 (66272 from Proteintech, 1:1000), occludin (BS-1495R from Bioss, 1:1000), (Zonula occludens-1) ZO-1 (TJP1, NBP1-85046 from Novus, 1:1000), TNFa (AF-510-NA from RD Systems, 1:500), and actin as loading control (ab6276 from Abcam, 1:5000). Secondary antibodies (1:4000) were IgGs conjugated with alkaline phosphatase (Sigma). Images were captured using a Hewlett Packard ScanJet 5300C and band intensities were quantified using AlphaImager 2200 software.

Band intensity was corrected by actin loading in the same blot. Data of quantification were expressed as percentage of the mean of control samples loaded in the same blot.

### 2.7. Analysis of Astrocytes and Microglia Activation 

Stained sections were scanned with a Pannoramic scanner and regions of interest were photographed with Pannoramic viewer software (3DHISTECH, Budapest, Hungary). Briefly, microglial activation was assessed by measuring the perimeter of Iba-1-stained cells in 10 randomly selected 56× fields per section, covering all areas of the stained slice, and results were expressed in micrometres. To analyse astrocyte activation, 10 fields (56×) were randomly photographed covering all areas of the stained slice and the total GFAP stained area was quantified.

### 2.8. Analysis of Lymphocytes and Macrophages Infiltration and Chemokines Expression in the Brain 

Brain infiltration was analyzed in all meningeal spaces. The number of foci (aggregates of 3 or more cells per field) was manually counted and the area of cerebellar meninges was measured using Image J. The results were expressed as number of foci/mm^2^. For the analysis of brain macrophage infiltration (with Iba-1 marker), all visible meninges in the slice were photographed, and for autoreactive CD4^+^/CX3CR1^+^ and Th17 CD4^+^/CCR6^+^ lymphocytes, 5 fields (63×) were randomly photographed covering all areas of the stained slice in the confocal microscope. Positive cells were manually counted and the length of meninges was measured using ImageJ. The results were expressed as cells/mm.

Analysis of CCL2, CCL20, and CX3CL1 in white matter and meninges of cerebellum was performed using ImageJ software. In white matter, 10 40× fields per rat were randomly photographed and the number of cells expressing CCL2, CCL20, or CX3CL1 was manually counted using the cell counter plugin of ImageJ. The area of the picture was measured and the results were expressed as cells/mm^2^. For the analysis of meninges, all areas with meninges in the slices were photographed and all stained cells in the meninges were manually counted. The length of meninges was measured. The results were expressed as cells/mm.

### 2.9. Analysis of TNF-a in White Matter and Purkinje Layer

Analysis of TNF-a in white matter and Purkinje layer was performed similarly. In white matter, 10 56× fields per rat were randomly photographed covering all areas of the stained slice, and the number of cells expressing TNF-a was manually counted. For the analysis of Purkinje neurons, 10 40× fields per rat were used. Purkinje neurons were manually selected using the freehand selection tool of the ROI manager function, and the mean intensity of TNF-a staining was recorded.

### 2.10. Analysis of Cellular Density 

The analysis of neuronal density in granular and Purkinje layers of the cerebellum was performed by ImageJ on H&E stained sections as in [17].

### 2.11. In Vivo Microdialysis and GABA and Glutamate Determination 

In vivo microdialysis in the cerebellum was performed as in [28]. Rats were anesthetized with isoflurane and a microdialysis guide was implanted in the cerebellum (AP −10.2, ML −1.6, and DV −1.2). After 48 h, a microdialysis probe was implanted in the freely moving rat. Probes were perfused (3 μL/min) with artificial cerebrospinal fluid (in mM): NaCl, 145; KCl, 3.0; CaCl2, 2.26; buffered at pH 7.4 with 2 mM phosphate. After a 2 h stabilization period, samples were collected every 30 min and stored at −80 °C until analysis of GABA and glutamate levels.

Extracellular basal concentration of GABA and glutamate was measured by HPLC-MS as in [28] in five consecutive microdialysis samples.

### 2.12. Analysis of Membrane Surface Expression of GABA and Glutamate Transporters

Membrane surface expression of GABA and glutamate transporters was analyzed as described by Cabrera-Pastor et al. [12]. Briefly, rats were sacrificed by decapitation and their cerebella were transferred into ice-cold Krebs buffer and transversally cut (400 μm) by vibratome. The slices were homogenized by sonication for 20 s. Samples treated with or without bis(sulfosuccinimidyl)suberate (BS3) crosslinker were analyzed by Western blot using anti-GAT-3 (274303 from Synaptic Systems, Goettingen, Germany, 1:500), anti-GAT-1 (ab426 from Abcam, 1:500), anti-GLAST (NB100-1869 from Novus Biological, 1:4000), and actin as loading control (ab6276 from Abcam, 1:5000). Band intensity for every protein was corrected by actin band intensity in the same blot. The surface expression of transporters was calculated as the difference between the intensity of the bands without BS3 (total protein) and with BS3 (non-membrane protein). Data of quantification were expressed as percentage of the mean of control samples in the same blot.

### 2.13. Analysis of Motor Coordination by Beam Walking or Rotarod Test 

The beam walking test was performed as in [28]. This test consists of a wood strip 20 mm in diameter and 1 m long, located 1 m above the ground. Rats have to cross the beam, and two observers count the number of times the rats slip off the beam. The average number of foot faults (slips) is a measure of incoordination.

The rotarod (Ugo Basile, Comerio, Italy) assesses the ability of rats to stay on a rotating drum with progressive acceleration. Two consecutive days before testing, each rat was placed on the rotarod (at constant speed), which was switched off for 3 min. During the test, the rotarod speed was increased from 4 to 40 rpm over 300 s. Motor incoordination was calculated as the mean latency to fall (2 trials) with a maximum cut-off of 600 s.

### 2.14. Statistical Analysis 

Data are expressed as mean ± SEM. All statistical analyses were performed using GraphPad Prism software v7.0. Data were tested for normality (Kolmogorov–Smirnov or D’Agostino and Pearson test) and for homogeneity of variances. When data did not pass the normality test, a nonparametric test was used: Kruskal–Wallis test, with Dunn’s test for multiple comparisons. When standard deviations (SDs) were not equal, Welch’s ANOVA test with Dunnett’s T3 multiple comparisons test was used. If the data showed normality and homogeneity of variances, statistical analysis was carried out using one-way ANOVA and Tukey’s multiple comparisons test or two-way ANOVA with repeated measures when appropriate, and Bonferroni’s multiple comparisons test. In case of comparisons of only two groups, unpaired Student *t*-test was used with Welch’s correction if SDs were not equal and Mann–Whitney test when data did not pass the normality test. A confidence level of 95% was considered as significant.

The number of animals used for each determination and the specific statistical test used, including its values, are indicated in figure and table captions.

## 3. Results

### 3.1. Characterization of Liver Damage in Rats Treated with CCl_4_ for Different Time Periods

**Figure 2:**
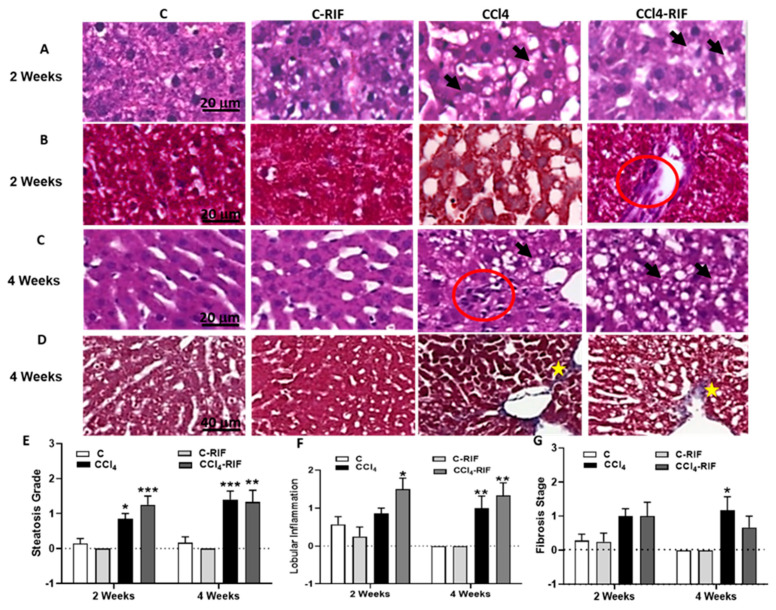
Hepatic damage: lipid deposition, lobular inflammation and fibrosis at 2 and 4 weeks. Representative images of (**A**,**C**) H&E and (**B**,**D**) Masson’s stains at 2 and 4 weeks of CCl_4_ treatment are shown. In rats treated with CCl_4_, increased lipid deposition was already present at 2 weeks (fat accumulation in liver is indicated with black arrows in (**A**,**C**); lobular inflammation (red circle in **C**) and collagen accumulation (yellow stars in **D**), only appeared at 4 weeks. No lesions were observed in control rats. Scale bar is 20 µm in (**A**–**C**) and 40 µm in (**D**). The grade of steatosis, inflammation and fibrosis was determined and is also represented in (**E**–**G**), respectively. Statistical analysis was carried out with Two-way ANOVA and Tukey’s multiple comparison test. Group effect was significant for the three parameters, but no significant effect for interaction or week of CCl_4_ administration was found. Statistic data were F(3,31) = 23.09, *p <* 0.0001 for steatosis, F(3,31) = 12.63, *p <* 0.0001 for inflammation and F(3,31) = 6.94, *p <* 0.001 for fibrosis differences between groups. Values are mean ± SEM of 6 rats per group. Values significantly different from control rats are indicated by asterisks. * *p <* 0.05, ** *p <* 0.01, *** *p <* 0.001. No significant differences between CCl_4_ and CCl_4_-RIF groups were found.

Progression of pathological features was assessed in the livers of rats treated with CCl_4_ for different time periods. Figure 2 shows hematoxylin–eosin staining to analyze steatosis and lobular inflammation and Masson staining to analyze hepatic fibrosis. After two weeks of CCl_4_ administration, rats showed fat accumulation in the liver (Figure 2E, black arrows), weak lobular inflammation (Figure 2F), and, in some cases, collagen fibers (Figure 2G). These changes are compatible with those observed in patients with mild steatohepatitis. At four weeks with CCl_4_, lobular inflammation and fibrosis were observed (Figure 2F,G, red circles and yellow stars, respectively). Treatment with rifaximin did not affect these alterations.

Inflammation in the liver was further characterized by measuring key cytokines at four weeks. As shown in Table 1, livers of rats with mild liver damage showed increased levels of TNF-α, TGF-β, IL-15, and IL-4, while the levels of IL-10 were reduced and IL-6, IL-17, and IL-18 remained unaltered. Rifaximin reversed the increase in IL-15, (*p <* 0.05 compared with CCl_4_ without rifaximin) but did not affect any of the other changes. In control rats, treatment with rifaximin increased TGF-β in the liver and tended to increase TNF-α (Table 1).

Liver damage was reflected in increased activity of alanine aminotransferase (ALT) in serum of rats with four weeks of CCl_4_, while aspartate aminotransferase (AST) and bilirubin were not significantly affected (Table 2). Treatment with rifaximin did not prevent the increase in ALT, indicating that it does not prevent liver damage.

### 3.2. Liver Damage Induces a Rapid Increase of Peripheral Inflammation and Alterations in Lymphocyte Populations in Blood but Not Hyperammonemia

Ammonia levels in blood were determined, but no significant changes were found after two or four weeks of CCl_4_ administration (38 ± 6 vs. 53 ± 12 µM in control and CCl_4_ rats, and 40 ± 6 vs. 56 ± 9 µM in control and CCl_4_ rats, respectively). Rifaximin did not affect blood ammonia levels in control or CCl_4_ rats. In this model of liver damage hyperammonemia does not appear until six weeks of CCl4 administration: at six weeks blood ammonia levels were 44 ± 8 µM in controls and 101 ± 19 µM in CCl4 rats. This agrees with previous reports [29]. A possible explanation is that, although liver function is already altered, and therefore detoxification of ammonia may be insufficient, is not yet so severe to compromise the detoxifying capacity of other systems in the body, such as muscle glutamine synthetase, maintaining blood ammonia levels in normal values.

Induction of liver damage was associated with a rapid increase in peripheral inflammation (Table 3). At one week, TNF-α, IL-6, IL-15, IFNγ, CX3CL1, and CCL5 increased in plasma (Table 3).

At two weeks, IL-6, IFN-γ and CCL5 returned to normal levels, while TNF-α, IL-15, and CX3CL1 remained increased, and IL-17 and CCL20 increased (Table 3). Treatment with rifaximin for one week normalized TNF-α, IL-15, and CCL20 but not IL-17 and CX3CL1 levels present at two weeks of liver damage (Table 4).

At four weeks, TNF-α and CX3CL1 remained increased. The pro-inflammatory IL-17, IL-15, and CCL20 returned to normal values, the anti-inflammatory IL-4 and TGF-β increased, and IL-10 decreased (Table 3). Treatment with rifaximin for two weeks normalized TNF-α and CX3CL1 levels and prevented the changes in IL-4 and TGF-β, but not IL-10 present at four weeks of liver damage (Table 4).

**Figure 4:**
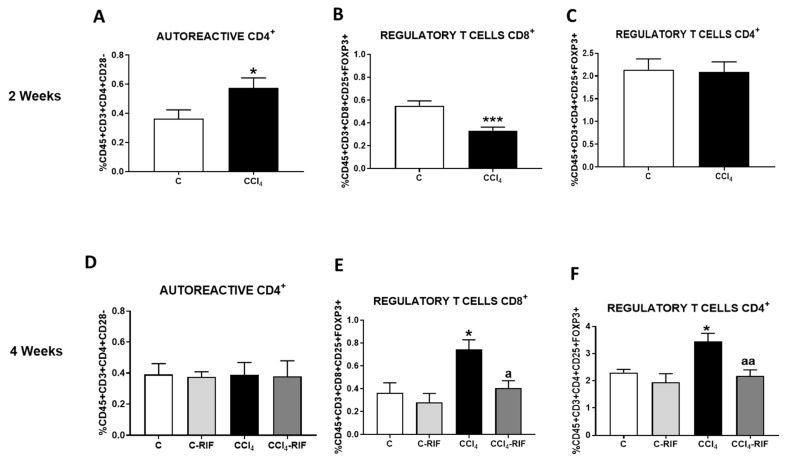
Analysis of autoreactive CD4^+^CD28^−^ and regulatory CD4^+^ and CD8^+^ T cells. (**A**,**D**) Autoreactive CD4^+^CD28^−^, (**B**,**E**) regulatory CD8^+^ T lymphocytes and (**C**,**F**) regulatory CD4^+^ T lymphocytes in whole blood at 2 and 4 weeks were analyzed by flow cytometry. Values are mean ± SEM of 8 rats per group. Student’s *t*-test was used at 2 weeks (*t* = 2.361, df = 13, *p* < 0.05 for autoreactive CD4^+^, *t* = 4.297, df = 28, *p* < 0.001 for regulatory CD8^+^) and one-way ANOVA with Tukey’s test for multiple comparisons at 4 weeks (F(3,24) = 0.0111, *p* > 0.05 for autoreactive CD4^+^, F(3,12) = 6.510, *p <* 0.01 for regulatory CD8^+^ and F(3, 15) = 7.61, *p <* 0.01 for regulatory CD4^+^ T cells). Values significantly different from control rats are indicated by asterisks and from CCl_4_ rats are indicated by a. * *p* < 0.05; *** *p* < 0.001; a *p <* 0.05; aa *p <* 0.01.

We analyzed lymphocyte populations in blood by flow cytometry. At two weeks of liver damage, autoreactive CD4^+^CD28^−^ lymphocytes increased and CD8^+^FoxP3^+^ regulatory T cells decreased (Figure 4A,B). The percentage of CD4^+^FoxP3^+^ regulatory lymphocytes were not affected at this time (Figure 4C). After four weeks of liver damage, both CD4^+^ and CD8^+^FoxP3^+^ regulatory T cells increased and autoreactive CD4^+^CD28^−^ cells returned to normal levels (Figure 4D–F). Rifaximin treatment for two weeks did not affect autoreactive CD4^+^CD28^−^ lymphocytes levels but prevented the increase of CD4^+^FoxP3 and CD8^+^FoxP3^+^ regulatory T cells in rats injected with CCl_4_ for four weeks (Figure 4E,F).

Gating strategy used in flow cytometry experiments and representative plots for each graph are shown in Figure S1.

### 3.3. Liver Damage-Induced Peripheral Inflammation Is Associated with the Infiltration of Monocytes–Macrophages and T Lymphocytes in Cerebellum and It Is Mediated by CCL2, CCL20, and CX3CL1 Chemokines and Increased BBB Permeability

**Figure 5:**
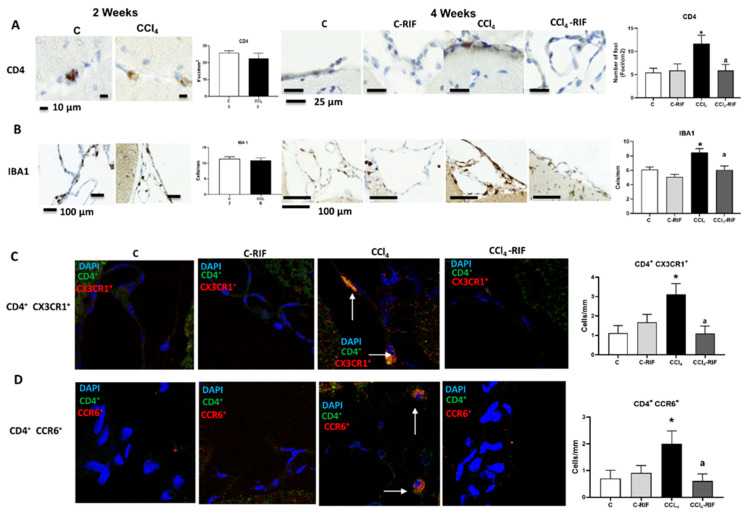
Rats with mild liver damage show immune cells infiltration in cerebellum at 4 weeks, which is prevented by rifaximin. Analysis of immune cells infiltration in cerebellum was performed at 4 weeks by immunohistochemistry using antibodies against (**A**) CD4, a marker of T lymphocytes (F(3,8) = 4.511, *p <* 0.01) and (**B**) IBA1, a marker of meningeal and perivascular macrophages (K-W(4,10) = 27.76, *p <* 0.0001). (**C**) Double immunofluorescence with anti-CD4 and anti-CX3CR1 as markers of autoreactive CD4^+^CD28^−^ T lymphocytes was performed and quantified (K-W(4,5) = 8.711, *p <* 0.05). (**D**) Double immunofluorescence with anti-CD4 and anti-CCR6 as marker of Th17 lymphocytes was performed and quantified (K-W(4,7) = 8.858, *p <* 0.05). Number of animals in each group was added under group names. One-way ANOVA with Tukey’s test (CD4^+^) and nonparametric Kruskal–Wallis (K-W statistic) with Dunn’s test (Iba1, CD4^+^/CX3CR1^+^ and CD4^+^/CCR6^+^) was performed to compare all groups. Values significantly different from control rats are indicated by asterisks and from CCl_4_ rats by a. * *p <* 0.05, a *p <* 0.05.

We then assessed the infiltration of monocytes–macrophages and T lymphocytes in cerebellum. At two weeks, no increase in monocyte and macrophage or T lymphocyte infiltration was observed (Figure 5A,B). However, at four weeks there was infiltration of CD4 lymphocytes (Figure 5A) and macrophages (from infiltrated monocytes) (Figure 5B) in cerebellar meninges.

Treatment with rifaximin completely prevented the infiltration of CD4^+^ lymphocytes and monocytes–macrophages (Figure 5A,B).

There was infiltration of CD4^+^ CX3CR1^+^ lymphocytes in CCl_4_ rats, suggesting infiltration of CD4^+^CD28^−^ autoreactive T lymphocytes, since the presence of CX3CR1 was reported to be a specific marker of CD4^+^CD28^−^ lymphocytes [15] (Figure 5C). Infiltration of CD4^+^/CCR6^+^ lymphocytes (Figure 5D) in cerebellum after four weeks of CCl_4_ administration was also observed, suggesting infiltration of Th17 T lymphocytes, since the surface expression of CCR6 is a marker of this linage [30]. Infiltration of both subtypes of lymphocytes was prevented by rifaximin.

Infiltration of monocytes–macrophages and lymphocytes into brain is promoted by chemokines that act chemoattractants, such as CCL2, CCL20, and CX3CL1.

**Figure 6:**
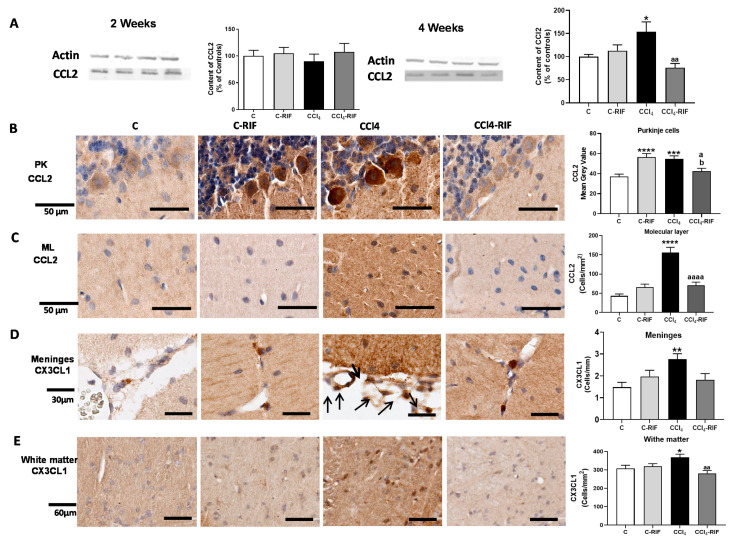

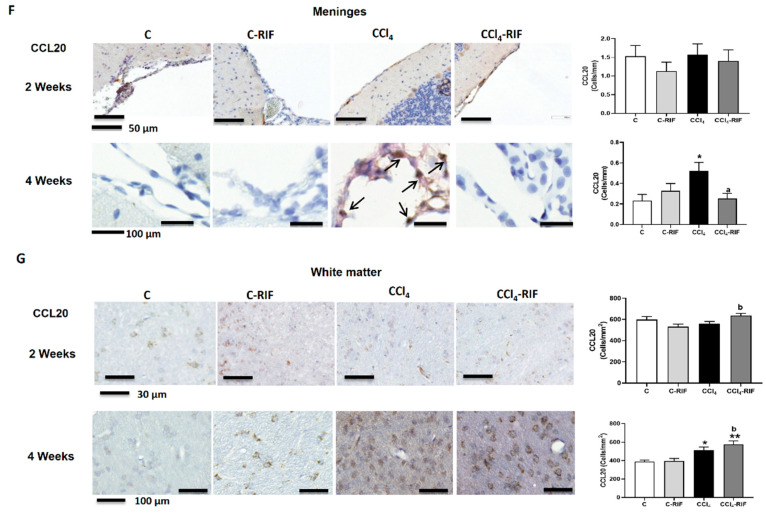
Mild liver damage induces the expression of CCL2, CX3CL1, and CCL20 in cerebellum. (**A**) Western blot analysis of CCL2 content at 2 and 4 weeks. Values are mean ± SEM of 9 rats per group. One-way ANOVA with Tukey’s test (F(3,13) = 9.998, *p* < 0.0001 for 4 weeks) (**B**,**C**) Representative IHQ images and analysis of CCL2 expression in Purkinje cells (**B**) and molecular layer at 4 weeks (**C**). Values are mean ± SEM of 3 rats per group. One-way ANOVA with Tukey’s test for Purkinje cells (F(3,13) = 9.998, *p <* 0.0001) and Welch’s ANOVA with Dunnett’s test for molecular layer (W(3,17) = 19.53, *p <* 0.0001) were performed to compare all groups. (**D**,**E**) Representative images of CX3CL1 expression in meninges and white matter and quantification at 4 weeks. Values are mean ± SEM of 5 rats per group. One-way ANOVA with Tukey’s test for white matter (F(3,18) = 5.063, *p <* 0.01) and nonparametric Kruskal–Wallis with Dunn’s test (K-W(4,21) = 11.48, *p <* 0.01) were performed to compare all groups. (**F**,**G**) Representative images of CCL20 expression in meninges (**F**) and white matter (**G**) and quantification at 4 weeks. Values are mean ± SEM of 4–5 rats per group. Welch’s ANOVA test with Dunnett’s test for white matter (W(3,14) = 8.261, *p* = 0.001) and nonparametric Kruskal–Wallis with Dunn’s test for meninges (K-W(4,11) = 9.412, *p <* 0.05) were performed to compare all groups. Values significantly different from control rats are indicated by asterisks, from CCl_4_ rats by a, and from C-RIF rats by b. * *p <* 0.05, ** *p <* 0.01, *** *p <* 0.001, **** *p <* 0.0001, a *p <* 0.05, aa *p <* 0.01, aaaa *p <* 0.0001, b *p <* 0.05.

At four weeks, but not at two weeks, the content of CCL2 was increased in the whole cerebellum (Figure 6A). Immunohistochemistry showed increased expression of CCL2, in both Purkinje neurons (Figure 6B) and glia in the molecular layer (Figure 6C). The CCL2 increase was prevented by rifaximin (Figure 6A,C).

Similar increases were observed for CX3CL1 at four weeks, in both meninges (Figure 6D) and white matter (Figure 6E). Rifaximin prevented the increase in both regions (Figure 6D,E).

The content of CCL20 was not affected at two weeks of liver damage in meninges (Figure 6F) or white matter (Figure 6G), but was remarkably increased at four weeks in both regions (Figure 6F,G). The increase in meninges, but not white matter, was prevented by rifaximin (Figure 6F,G).

**Figure 7:**
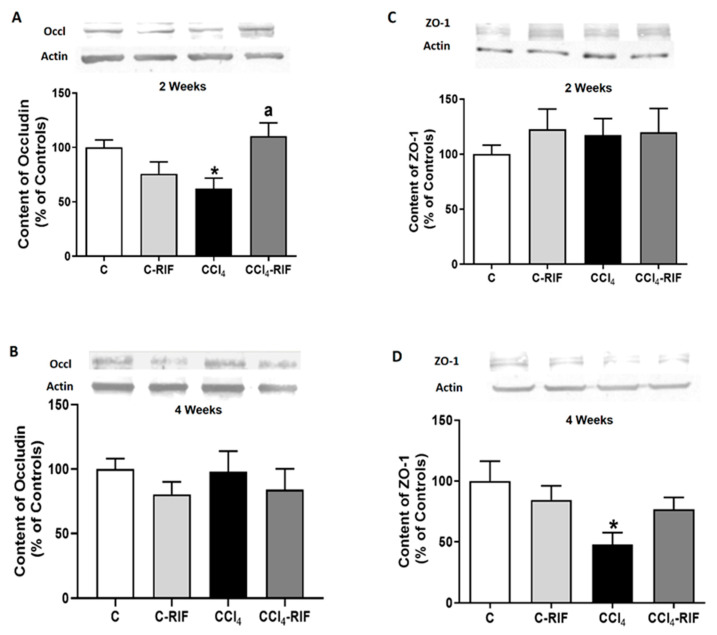
Expression of tight junction proteins in cerebellum of rats with mild liver damage. Expression of (**A**,**B**) occludin and (**C**,**D**) ZO-1 at 2 and 4 weeks was analyzed by Western blot. Values are mean ± SEM of 10–12 rats per group. One-way ANOVA with Tukey’s test was performed for occludin at 2 weeks (F(3,27) = 4.99, *p <* 0.01) and 4 weeks (F(3,47) = 0.611, *p* > 0.05) and for ZO-1 at 2 weeks (W(3,23) = 0.715, *p* > 0.05) and 4 weeks (F(3,43) = 3.698, *p <* 0.05). Values significantly different from control rats are indicated by asterisks and from CCl_4_ rats by a. * *p <* 0.05; a *p <* 0.05.

To assess whether the infiltration of lymphocytes and macrophages was associated with permeabilization of the blood–brain barrier (BBB), we analyzed the content of occludin and Zonula occludens-1 (ZO-1). Occludin content was reduced in cerebellum of rats at two weeks of liver damage (Figure 7A), but not at four weeks (Figure 7B). ZO-1 was not reduced at two weeks (Figure 7C) but was reduced at four weeks (Figure 7D). These changes in some tight junction proteins suggest mild BBB permeabilization at both two and four weeks of liver damage. Treatment with rifaximin prevented the reduction of occludin. In the case of ZO-1, there was a partial prevention of the decrease, which did not reach statistical significance. However, ZO-1 reached similar levels than control rats treated with rifaximin, that tended to reduce ZO-1 levels in control rats (Figure 7A,D). Altogether, these changes suggest an improvement of tight junctions in the BBB. This rifaximin effect must be indirect, mediated by effects on peripheral inflammation, since rifaximin is not absorbed in the intestine.

### 3.4. Two Weeks of Liver Damage Are Enough to Induce Neuroinflammation in the Cerebellum of Rats Treated with CCl_4_

**Figure 8:**
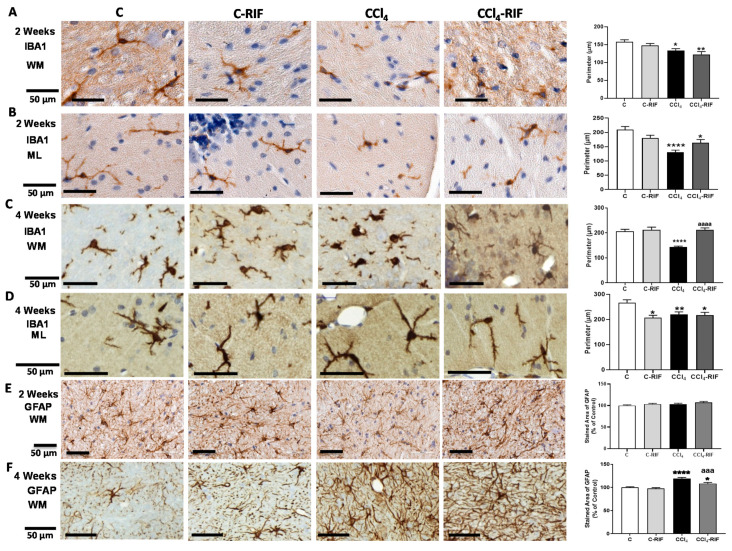
Analysis of microglia and astrocytes activation in cerebellum of rats with mild liver damage. (**A**,**B**) Representative images and analysis of activation state of microglia in white matter (WM) and molecular layer (ML) at 2 weeks. One-way ANOVA with Tukey’s test was performed to compare all groups (WM F(3,6) = 5.283, *p <* 0.01; ML F(3,5) = 12.43, *p <* 0.0001). (**C**,**D**) Representative images and analysis of activation state of microglia in white matter and molecular layer at 4 weeks. Kruskal–Wallis (K-W statistic) with Dunn’s test was performed. For WM, K-W(4,8) = 45.34, *p <* 0.0001 and for ML, K-W(4,6) = 15.1, *p <* 0.01. (**E**,**F**) Representative images and analysis of activation state of astrocytes in white matter at 2 and 4 weeks. Values are mean ± SEM of 3–5 rats per group. One-way ANOVA with Tukey’s test was performed to compare all groups at 2 weeks (F(3,14) = 1.536, *p* > 0.05) and Welch’s ANOVA was performed at 4 weeks (W(3,11) = 23.91, *p <* 0.0001). Values significantly different from control rats are indicated by asterisks and from CCl_4_ rats by a. * *p <* 0.05, ** *p <* 0.01, **** *p <* 0.0001, aaa *p <* 0.001, aaaa *p <* 0.0001.

We assessed the activation of microglia and/or astrocytes. Microglia were already activated at two weeks. The perimeter of microglia was reduced in both white matter (Figure 8A) and molecular layer (Figure 8B), reflecting microglial activation. Treatment with rifaximin for one week did not prevent microglia activation at two weeks of liver damage (Figure 8A,B). At two weeks of liver damage, activation of astrocytes was not observed (Figure 8E).

At four weeks, microglia remained activated in white matter (Figure 8C) and molecular layer (Figure 8D). Rifaximin reversed microglia activation in white matter (Figure 8C) but not in the molecular layer (Figure 8D).

Astrocytes were also activated in white matter at four weeks and the area covered by GFAP was increased. Treatment with rifaximin for two weeks prevented astrocyte activation (Figure 8F).

**Figure 9:**
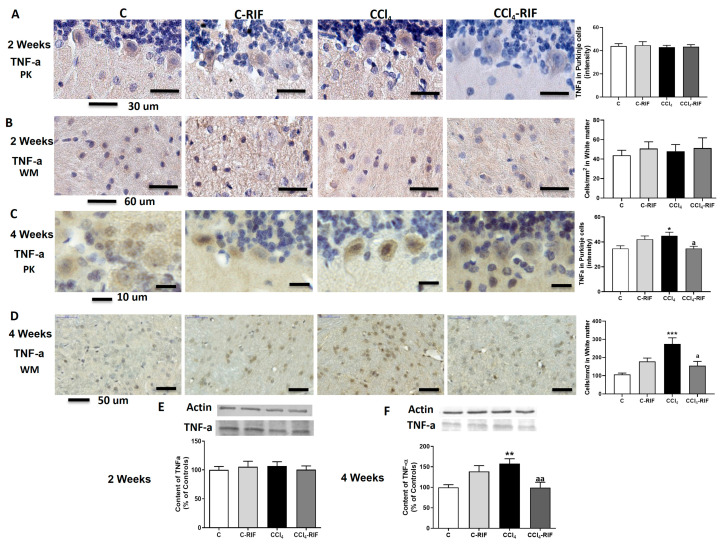
Expression of TNF-α in cerebellum of rats with liver damage. (**A**,**B**) Representative images and analysis of TNF-a expression in Purkinje neurons and white matter of cerebellum at 2 weeks. (**C**,**D**) Representative images and analysis of TNF-α expression in Purkinje neurons and white matter of cerebellum at 4 weeks. Values are mean ± SEM of 3–4 rats per group. Welch’s ANOVA test with Dunnett’s test for multiple comparisons was performed for Purkinje cells at 2 weeks (W(3,13) = 2.938, *p <* 0.05) and white matter at 4 weeks (W(3,10) = 11.03, *p <* 0.0001). Kruskal–Wallis with Dunn’s test was performed for white matter at 2 weeks (K-W(4,8) = 0.569, *p* > 0.05) and Purkinje cells at 4 weeks (K-W(4,5) = 12.02, *p <* 0.01). Content of TNF-α in cerebellum at (**E**) 2 and (**F**) 4 weeks was also quantified by Western blot. Values are mean ± SEM of 10–16 rats per group. One-way ANOVA with multiple comparisons with Tukey’s test was performed (F(3,15) = 5.927, *p <* 0.01 at 4 weeks; F(3,16) = 0.219, *p* > 0.05 at 2 weeks). Values significantly different from control rats are indicated by asterisks and from CCl_4_ rats by a. * *p* < 0.05, ** *p* < 0.01, *** *p* < 0.001, a *p* < 0.05, aa *p* < 0.01.

We then analyzed TNF-α. At two weeks, no change in TNF-α was observed in Purkinje neurons (Figure 9A), white matter (Figure 9B), or whole cerebellum (Figure 9E).

However, at four weeks of liver damage, TNF-α increased in Purkinje neurons (Figure 9C), white matter (Figure 9D), and whole cerebellum (Figure 9F). Treatment with rifaximin prevented TNF-α increase (Figure 9C,D,F).

**Figure 10:**
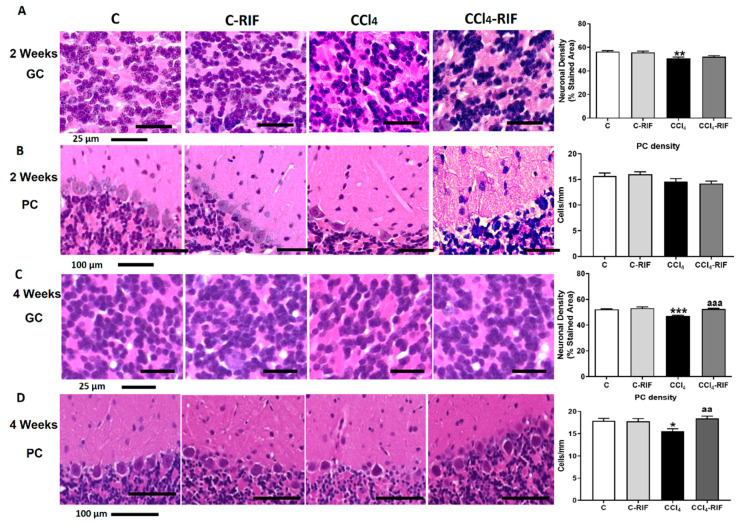
Rats with mild liver damage show neuronal loss in granular layer from 2 weeks and Purkinje neurons at 4 weeks. Representative images and neuronal density quantification of granular neurons (**A**,**C**) and Purkinje cells (**B**,**D**) of cerebellum stained with H&E. Values are mean ± SEM of 4–6 rats per group. One-way ANOVA with Tukey’s test for granule neurons (F(3,18) = 13.25, *p <* 0.0001) and Purkinje cells (F(3,16) = 2.425, *p* > 0.05) at 2 weeks was performed. Kruskal–Wallis with Dunn’s test for granule neurons (K-W(4,11) = 38.84, *p <* 0.0001) and Purkinje neurons (K-W(4,19) = 10.47, *p <* 0.05) at 4 weeks was performed to compare all groups. Values significantly different from control rats are indicated by asterisks and from CCl_4_ rats by a. * *p* < 0.05, ** *p* < 0.01, *** *p* < 0.001, aa *p* < 0.01, aaa *p* < 0.001.

We assessed whether neuroinflammation was associated with neuronal loss. At two weeks, there was a decrease in the cellular density of granular neurons (Figure 10A) but not Purkinje neurons (Figure 10B). At four weeks, the neuronal loss in granular layer increased even more but was completely stopped by treatment with rifaximin for two weeks (Figure 10C). At this time, there was a loss of Purkinje neurons, which was prevented by two weeks of rifaximin (Figure 10D).

### 3.5. Neuroinflammation Mediates Altered Neurotransmission and Motor Incoordination in Rats with Liver Damage. Reversal by Rifaximin

We assessed whether neuroinflammation was associated with altered neurotransmission and motor incoordination.

**Figure 11:**
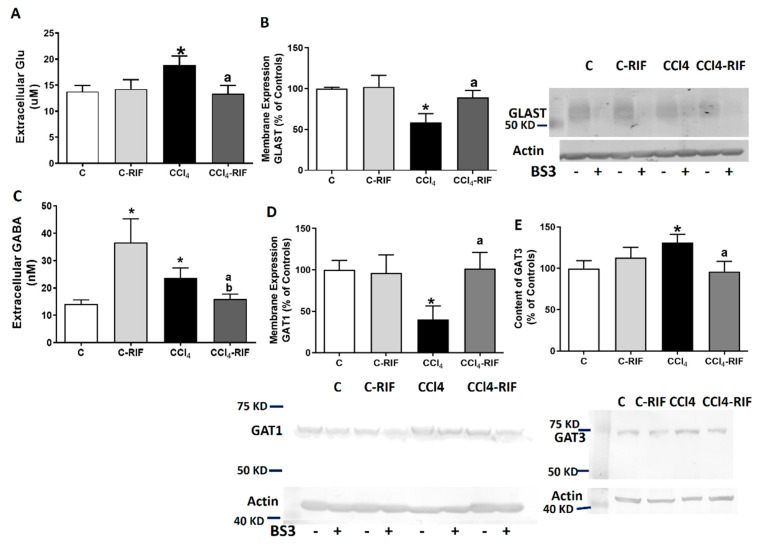
Analysis of extracellular levels of GABA and Glutamate and membrane expression of their transporters. Extracellular levels of (**A**,**C**) GABA and glutamate were analyzed by microdialysis at 4 weeks. Membrane expression of (**B**) glutamate transporter GLAST and (**D**,**E**) GABA transporters GAT1 and GAT3 were analyzed using BS3 cross-linker. Values are expressed as percentage of control rats and are mean ± SEM of 12 rats per group. One-way ANOVA with Tukey’s test for GAT-1 (F(3,27) = 4.41, *p <* 0.05), GAT-3 (F(3,41) = 3.27, *p <* 0.05) and glutamate (F(3,36) = 3.00, *p <* 0.05) and Welch’s ANOVA with Dunnett’s test for GABA (W(3,19) = 3.67, *p <* 0.05) and GLAST (W(3,13) = 12.07, *p <* 0.001) were performed to compare all groups. Values significantly different from control rats are indicated by asterisks, from CCl_4_ rats by a, and from C-RIF rats by b. * *p <* 0.05, a *p <* 0.05, b *p <* 0.05.

Extracellular glutamate levels were increased in rat cerebellum after two weeks of CCl_4_ administration (Figure 11A), and this was associated with reduced membrane expression of the glutamate transporter GLAST (Figure 11B). Both effects were normalized by rifaximin (Figure 11A,B).

Extracellular GABA levels were also increased, and normalized by rifaximin (Figure 11C). The increase in GABA was associated with reduced membrane expression of the neuronal GABA transporter GAT1 (Figure 11D) and an increase of the astrocytic transporter GAT3 (Figure 11E). Treatment with rifaximin normalized membrane expression of both GAT1 and GAT3 (Figure 11D,E).

**Figure 12:**
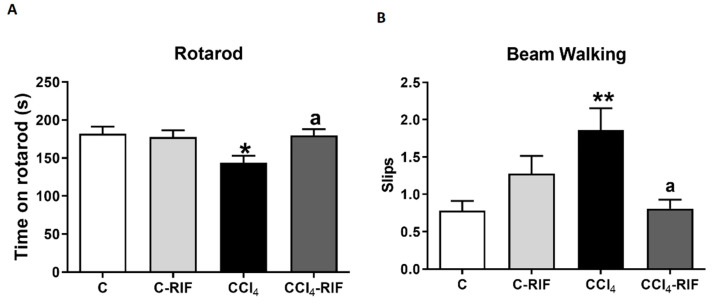
Mild liver damage induces motor in-coordination in rats which is prevented by rifaximin. Motor coordination was analyzed by (**A**) rotarod at 3 weeks and (**B**) beam walking at 4 weeks. Values are mean ± SEM of 10–14 rats per group. One-way ANOVA with Tukey’s test for rotarod (F(3,43) = 3.327, *p* < 0.05) and Welch’s ANOVA with Dunnett’s test for beam (W(3,26) = 4.851, *p* < 0.01) were performed to compare all groups. Values significantly different from control rats are indicated by asterisks and from CCl4 rats by a. * *p* < 0.05, ** *p* < 0.01, a *p* < 0.05.

Rats with mild liver damage showed impaired motor coordination in the rotarod, remaining in the rod for less time than control rats. Treatment with rifaximin restored motor coordination in these rats (Figure 12A). Similar results were obtained in the beam walking test. Rats with mild liver disease showed more slips than control rats, and treatment with rifaximin restored motor coordination (Figure 12B).

## 4. Discussion

**Figure 13:**
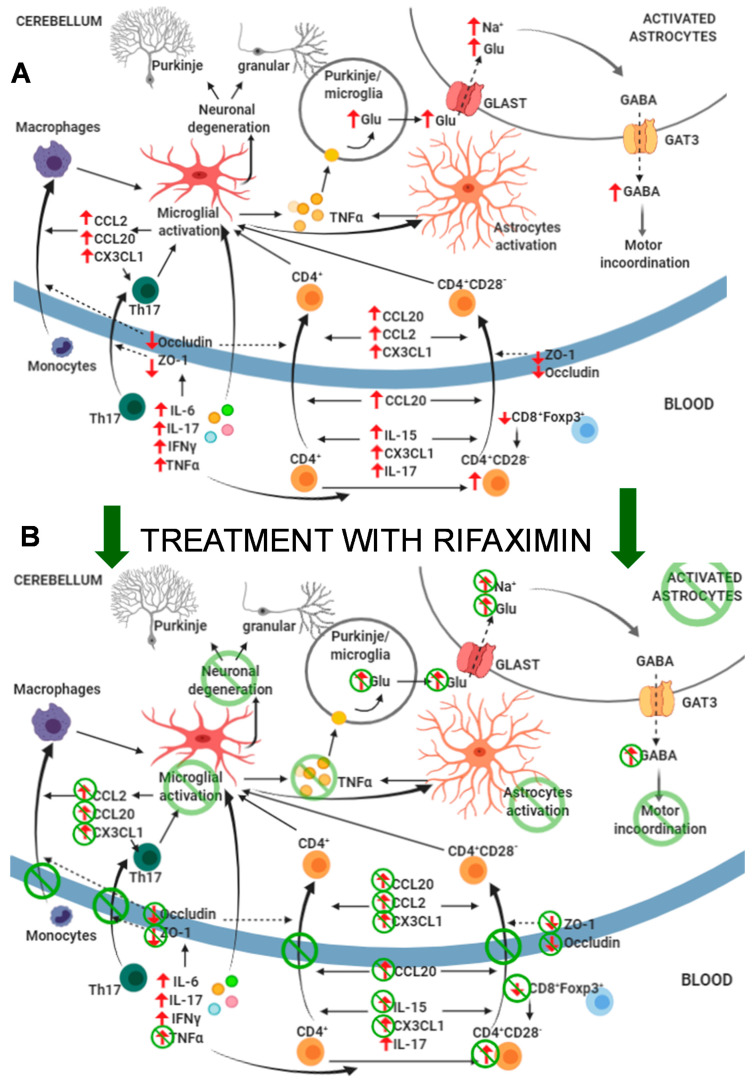
Proposed events involved in the effects of mild liver damage on cerebellum and motor coordination and effects of rifaximin. (**A**) Mild liver damage induces a pro-inflammatory environment in peripheral blood with increased autoreactive CD4^+^CD28^−^ lymphocytes and TNF-α, IFN-γ, IL-6, and IL-17, which induce microglia activation at 2 weeks of liver damage. This induces CCL2, CCL20, and CX3CL1 expression in cerebellum, which, together with increased CCL20, IL-15, CX3CL1, and IL-17 in plasma and altered BBB due to reduced occludin and ZO-1, promotes infiltration of CD4^+^CD28^−^ and Th17 lymphocytes and monocytes–macrophages at 4 weeks. This potentiates neuroinflammation and activation of microglia and astrocytes and increases formation and extracellular concentration of glutamate in cerebellum. Increased uptake of glutamate by activated astrocytes results in reversed function of GABA transporter GAT3 and increased extracellular levels of GABA, which leads to motor incoordination. (**B**) Treatment with rifaximin prevents most changes in peripheral inflammation, normalizing levels of autoreactive CD4^+^CD28^−^ lymphocytes, TNF-α, CCL20, IL-15, and CX3CL1. This prevents permeabilization of the BBB and reduces microglia activation and increased CCL2, CCL20, and CX3CL1 in cerebellum. As a consequence, infiltration of monocytes and lymphocytes is also prevented, as well as subsequent potentiation of microglia and astrocyte activation. This leads to normalization of extracellular levels of glutamate and GABA in cerebellum and restoration of motor coordination.

This study shows that mild liver damage is associated with neuroinflammation and altered neurotransmission in the cerebellum and motor incoordination in rats. These alterations are a consequence of increased systemic inflammation, which causes an infiltration of CD4 T-lymphocytes and macrophages into the brain. It is also shown that early treatment with rifaximin prevents the infiltration of lymphocytes and macrophages, reduces neuroinflammation, and restores neurotransmission and motor coordination in these rats. The main results are summarized in Figure 13.

During the first two weeks of liver damage, rats showed an increase in proinflammatory IFNγ, TNF-α, and IL-6 (acute-early phase), and later also an increase in IL-17 (acute-late phase). These changes agree with those observed in patients with steatohepatitis [31].

After four weeks, an increase in anti-inflammatory IL-4 and TGF-β and a decrease in IL-10 were observed. All of these factors are involved in mechanisms aimed at suppressing the synthesis of proinflammatory and fibrogenic cytokines [31].

Inflammatory stress induced by IL-6, TNF-α, and/or IL-17 leads to a loss of integrity of the BBB, with decreased tight junction proteins such as occludin and ZO-1 [32,33]. In rats with liver damage induced by CCl_4_ administration, there was a rapid increase in TNF-α and IL-6 at one week, followed by an increase in TNF-α and IL-17 at two weeks, which would be responsible for the decrease in occludin and ZO-1, and BBB reduced integrity in cerebellum at two and four weeks, which would facilitate the infiltration of CD4 lymphocytes and macrophages at four weeks. However, future research is necessary to study in more detail the mechanism of BBB permeabilization in our model, including analysis of adhesion molecules such as intercellular adhesion molecule-1 (ICAM-1) or vascular adhesion molecule-1 (VCAMP-1) on endothelial cells and other relevant tight junctions proteins as claudin 5.

The prevention of most changes in peripheral inflammation by rifaximin would explain why it prevents occludin and ZO-1 reduction, and CD4 lymphocytes and macrophages infiltration.

During the progression of liver damage in fatty liver disease, the accumulation of fat in hepatocytes triggers oxidative stress, which induces the production and release of TNF-α and TGF-β by Kupffer cells [34,35]. We observed increased levels of both TGF-β and TNF-α in liver at four weeks of CCl_4_ administration, which was not reversed by rifaximin, indicating that rifaximin does not prevent activation of Kupffer cells. In fact, the levels of TGF-β were significantly increased in the liver of control rats treated with rifaximin, and TNF-α also tended to increase, suggesting mild activation of Kupffer cells by rifaximin treatment.

Kupffer cells in liver tissues of patients with steatohepatitis show reduced levels of RORa, which contributes to increasing the production of TNF-α but also reducing the formation of IL-10 in Kupffer cells [36]. The activation of Kupffer cells would thus also contribute to the reduced content of IL-10 in livers of CCl_4_ rats at four weeks. As is the case for TNF-α and TGF-β, this effect was not prevented by rifaximin, further supporting the idea that rifaximin does not prevent Kupffer cells activation.

In addition, monocyte-derived macrophages infiltrate to the liver and release pro-inflammatory cytokines that activate other immune cells [36,37]. Infiltrating monocytes are the main producers of IL-15 in the liver [38]. We found an increase in IL-15 in liver of rats at four weeks of CCl4, which, in contrast to the effects on TNF-α or TGF-β, was prevented by rifaximin treatment. This suggests that rifaximin treatment would reduce the infiltration of monocytes into the liver or the production of IL-15 by these monocytes.

In steatohepatitis, the liver shows increased levels of activated NKT cells [39]. Activated IL-17-secreting NKT cells were dominant in liver during the beginning of the steatosis phase, whereas IL-4/IL-13-secreting iNKT cells were prevalent later in the disease [40]. We found increased levels of IL-4 but not IL-17 in liver of rats at four weeks of CCl_4_. It is likely that the increased levels of IL-4 would be produced by activated NKT cells. This effect is not prevented by rifaximin, suggesting that rifaximin does not prevent activation of NKT cells.

Infiltration of monocytes–macrophages and T lymphocytes is promoted by certain cytokines that act as chemoattractants. CCL2 and CX3CL1 promote recruitment of peripheral monocytes [41,42,43,44] and T lymphocytes [42]. CCL20 promotes recruitment of different subtypes of CD4 lymphocytes, including Th1, Th17, autoreactive, and Tregs [30,45,46].

IL-17 promotes CCL2 [47] and CCL20 [48] expression, and IL-15 is a potent T cell chemoattractant that allows lymphocyte infiltration into the brain [49].

At two weeks of liver damage, CCL20, CX3CL1, IL-15, and IL-17 were increased in plasma, providing an environment that promotes recruitment of monocytes and lymphocytes into the brain. At four weeks, CX3CL1 and IL-15 remained increased in plasma; moreover, CCL2, CCL20, and CX3CL1 increased in cerebellum. These changes, together with the BBB permeabilization (reduction of occludin and ZO-1), would promote the recruitment and infiltration of monocytes–macrophages and CD4 lymphocytes into the cerebellum of rats with mild liver damage. This infiltration occurs between the second and fourth week of liver damage, when the environment is more appropriate for it.

The decrease in occludin at two weeks but not at four weeks of CCl_4_ administration and the decrease in ZO-1 expression only at four weeks may be due to a different modulation. Different cytokines, which show changes in plasma levels between two and four weeks, can affect the expression of these two proteins differently. Other studies have also suggested different modulation of the expression of these two proteins in the intestinal barrier. Guo et al. [50] found an increase in occludin associated with a reduction in IL-10, without affecting the expression of ZO-1, in the intestinal barrier. The reduction of IL-10 in CCl4 rat plasma at four weeks could explain that occludin is no longer decreased at this time but is at two weeks. Moreover, in the colon of rats with alcohol-induced steatohepatitis, occludin was found to be decreased but not the expression of ZO-1 [51], and conversely, heat stress affected the intestinal expression of ZO-1 but not that of occludin [52].

Two weeks of treatment with rifaximin reduced some of these changes at four weeks of liver damage, normalizing CX3CL1 and IL-15 in plasma and CCL2, CCL20, and CX3CL1 in cerebellum. This would contribute to preventing immune cell infiltration into cerebellum. One week of treatment with rifaximin afforded partial protection at two weeks of liver damage, reducing CCL20 and IL-15 (but not CX3CL1 and IL-17) in plasma and occludin expression in cerebellum.

These effects of rifaximin, together with the prevention of changes in CD8^+^FoxP3^+^ regulatory T cells and CD4^+^CD28^−^ lymphocytes, would explain why it prevents monocytes and lymphocytes infiltration in cerebellum of rats with mild liver damage. The results also suggest that rifaximin treatment for one week is not enough to completely induce all of the beneficial effects and that at least two weeks are required.

Liver damage in rats is associated with increased CD4^+^CD28^−^ lymphocytes in blood and infiltration of these lymphocytes and Th17 CD4 lymphocytes in cerebellum. This reproduces the effects found in patients with steatohepatitis, who also show infiltration of CD4^+^CD28^−^ and Th17 CD4 lymphocytes in cerebellum [17]. Autoreactive CD4^+^CD28^−^ and Th17 lymphocyte activation are also increased in the blood of cirrhotic patients with MHE [13] and in other inflammatory syndromes [53]. The loss of CD28 in CD4 lymphocytes is a consequence of persistent immune activation. Prolonged exposure to TNF-α reduces CD28 expression in T cells by reducing its transcription [53]. This suggests that sustained activation by TNF-α would contribute to the increased CD4^+^CD28^−^ lymphocytes in rats with mild liver damage. As rifaximin normalizes TNF-α in blood, this would contribute to the normalization of CD4^+^CD28^−^ lymphocyte content in rats with liver damage by rifaximin.

CD8^+^FoxP3^+^ regulatory T cells inhibit CD4^+^CD28^−^ cells proliferation and IFN-γ and IL-17 secretion [54]. At two weeks of liver damage, the amount of CD8^+^FoxP3^+^ regulatory T cells is reduced in blood, allowing proliferation and an increase in autoreactive CD4^+^CD28^−^ cells, which produce IFN-γ and IL-17 [55]. As liver damage progresses, the amount of CD8^+^FoxP3^+^ regulatory T cells increases and suppresses autoreactive CD4^+^CD28^−^ cells proliferation, which returns to normal levels at four weeks, together with IFN-γ and IL-17. Rifaximin normalizes the changes in CD8^+^FoxP3^+^ regulatory T cells.

CD4^+^CD28^−^ and activated Th17 cells express cytotoxic molecules, including perforin, granzymes, and adhesion molecules, which enhances their capacity to infiltrate tissues [15,56], including brains of patients with multiple sclerosis [57]. Once activated, CD4^+^CD28^−^ and Th17 lymphocytes can migrate into the brain and initiate a pro-inflammatory response [56,57].

Activation of microglia in cerebellum precedes the infiltration of lymphocytes and macrophages, which is observed already at two weeks of liver damage. A main mechanism by which peripheral inflammation may activate microglia independently of immune cells infiltration is activation by peripheral interleukins (TNF-α, IL-1b, IL-6) of their receptors in endothelial cells, triggering the release of inflammatory factors into the brain [58]. It is likely that this is the initial mechanism by which liver damage triggers microglia activation. Subsequent infiltration of macrophages and T lymphocytes potentiates and maintains sustained neuroinflammation in cerebellum of rats with liver damage.

This initial microglial activation may induce the expression of chemokines such as CCL2, CCL20, and CX3CL1, which promote the recruitment of peripheral monocytes and lymphocytes [41,43]. In mice with bile duct ligation, peripheral TNF-α signaling stimulates microglia to produce CCL2, which promotes the infiltration of monocytes into cerebral cortex. The infiltration does not occur in mice lacking CCL2 [41].

In agreement with the above report, rats with mild liver damage showed microglia activation and increased CCL2 levels in cerebellum at four weeks, which would trigger the recruitment of monocytes into the cerebellar meninges. These infiltrated macrophages encounter an inflammatory environment and contribute to the ongoing neuroinflammation and sustained activation of microglia [42,43].

Treatment with rifaximin reversed microglia activation in white matter but not in the molecular layer, and completely prevented the increase in CCL2, which, together with the prevention of changes in peripheral immune system and TNF-α, would lead to the observed prevention of monocyte infiltration.

Once infiltrated, CD4 lymphocytes and macrophages contribute to promoting neuroinflammation and activation of microglia [42,57]. Prevention by rifaximin of the infiltrating peripheral immune system cells contributes to reducing neuroinflammation and restoring neurotransmission and motor coordination in rats with mild liver damage. We also analyzed the mechanisms by which neuroinflammation induces motor incoordination in rats with mild liver damage. In rats with chronic hyperammonemia, neuroinflammation in cerebellum induces motor incoordination by increasing extracellular glutamate. Increased glutamate uptake and astrocytes activation lead to reversed function of the GABA transporter GAT3. This, together with its enhanced membrane expression, leads to the release and increased levels of extracellular GABA, which leads to motor incoordination [9].

We therefore analyzed this process. Rats with mild liver damage showed increased levels of extracellular glutamate and GABA in cerebellum, associated with reduced membrane expression of the glutamate transporter GLAST and the neuronal GABA transporter GAT1 and increased membrane expression of the astrocytic GABA transporter GAT3. This indicates that, in rats with mild liver damage, neuroinflammation induces motor incoordination by increasing extracellular GABA through the above process.

Treatment with rifaximin reduces neuroinflammation and normalizes extracellular glutamate, and membrane expression of the glutamate and GABA transporters and extracellular GABA. This leads to restoration of motor coordination in rats with mild liver damage.

We show that rifaximin is able to prevent or reverse all of the above alterations by reducing the pro-inflammatory environment in peripheral inflammation and preventing permeabilization of the BBB and T lymphocyte and macrophage infiltration, along with the associated neuroinflammation and alterations in GABAergic neurotransmission, and restoring motor coordination.

Rifaximin is a non-absorbable antibiotic that was approved as treatment for the prevention of HE in the USA in 2010 [59]. Rifaximin reduces the risk of overt HE recurrence and mortality [59] and also improves cognitive and motor function in a large percentage of cirrhotic patients with MHE [23].

However, the mechanisms by which rifaximin induces beneficial effects on brain function remain unclear. There are some reports in the literature on the beneficial effects of rifaximin on the peripheral immune system [59,60], while other reports show minor effects of rifaximin on peripheral inflammation [61]. Rifaximin alters the microbiota, and these alterations are transmitted to the blood through the gut–immune system connection. The effects of rifaximin on liver and brain would be mediated by alterations in immune molecules in circulating blood, as is proposed in the gut–liver–brain axis hypothesis [61,62]. According to this hypothesis, Mangas-Losada et al. [23] showed that rifaximin reduced peripheral inflammation in cirrhotic patients with improved MHE but not in those who did not improve. The results shown here support the alteration of the peripheral immune system by rifaximin, but also show effects on organs such as liver and brain, both in control rats and rats with hepatic damage. However, the anti-inflammatory effect of rifaximin in the liver of rats with hepatic damage was less remarkable than in the cerebellum. This support the idea that the effects of rifaximin on neurological alteration in MHE is due to the changes in peripheral inflammation, but rifaximin does not have a main effect on liver damage, as inflammation, fibrosis, and transaminase activity in serum are not prevented by rifaximin.

Rifaximin treatment induces significant effects in control rats: it increases CCL2 in Purkinje neurons, induces microglia activation (Figure 8), and increases GABA in the cerebellum. Rifaximin increases TGF-β levels in both liver and plasma, likely by mildly activating Kupffer cells. Moreover, after only one week of treatment, rifaximin increased CCL20 in plasma of control rats.

These data suggest that rifaximin alters the peripheral immune system in normal conditions in control rats, and this would also lead to immunological alterations in the liver and cerebellum, resulting in some alterations in neurotransmission, including an increase in extracellular GABA, which could cause the mild, not significant, increased number of slips in the beam walking test. The increased CCL2 in neurons may contribute to activating microglia in cerebellum [63,64]. It should be taken into account that the microbiota may be altered in rats with liver damage by injection of CCl_4_ [59,65]. This suggests that the effects of rifaximin on the microbiota may be different in control rats than in rats with liver damage and therefore induce different changes in the immune system. This could explain the differential effects of rifaximin on the plasma levels of CCL20 and TGF-β, which are increased in control rats but decreased in rats with mild liver damage.

## 5. Conclusions

The above data, together with the results reported here, suggest that MHE is triggered by a shift of peripheral inflammation to a pro-inflammatory environment, with increased levels of TNF-α, IL-6, IL-15, IL-17, CCL20, and CX3CL1, that promotes infiltration of monocytes and lymphocytes in the cerebellum. Rifaximin prevents some of these alterations, mainly the initial increase of TNFα and IL-15 and the increase in the main chemokines involved in the infiltration process in CCl_4_ rats. Therefore, rifaximin prevents the infiltration of peripheral immune cells into the brain and the subsequent enhancement of neuroinflammation, changes in neurotransmission, and cognitive and motor impairment.

The results reported here support the previously reported idea that patients with NASH may already have cognitive and motor impairment before reaching cirrhosis [18], and that early treatment of these patients with rifaximin would prevent the induction of these neurological alterations.

## Figures and Tables

**Figure 3:**
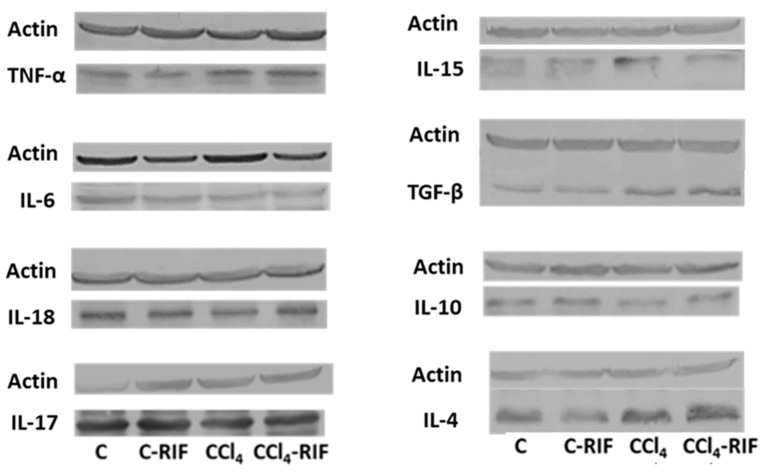
Representative bands from western blots of liver cytokines corresponding to data described in Table 1.

**Table 1:** Liver inflammation: content of cytokines. Changes in liver cytokines at 4 weeks of treatment with CCl_4._ The levels of all cytokines was analyzed by western blot. Data are the mean ± SEM of the indicated number of rats and are given as percentage of control rats. Six rats per group were used for this analysis. One-way ANOVA and Bonferroni’s or Dunnett’s tests for multiple comparisons were performed. Values significantly different from control rats are indicated by asterisks, * *p <* 0.05; statistical differences between CCl_4_ and CCl_4_-RIF are indicated by “a”, *p <* 0.05. Representative bands of liver cytokines western blots are shown in Figure 3.

Content of Cytokines in Liver (% of Control)	C-RIF	CCl_4_	CCl_4_-RIF
TNF-α	137 ± 22	150 ± 11 *	140 ± 26
IL-6	109 ± 9	98 ± 10	97 ± 7
IL-18	92 ± 9	99 ± 7	102 ± 7
IL-17	70 ± 9	117 ± 35	65 ± 12
IL-15	99 ± 9	151 ± 15 *	81 ± 15 ^a^
TGF-β	177 ± 14 *	169 ± 23 *	218 ± 14 *
IL-10	87 ± 5	72 ± 7 *	80 ± 6
IL-4	116 ± 13	163 ± 15 *	186 ± 25

**Table 2:** Hepatic enzyme activities in serum. Transaminase activity and bilirubin levels were assessed as indicators of liver injury. Six rats per group were used for this analysis. One-way ANOVA and Bonferroni’s or Dunn’s tests for multiple comparisons were performed. Values significantly different from control rats are indicated by asterisks, * *p <* 0.05.

	C	C-RIF	CCl_4_	CCl_4_-RIF
ALT (U/L)	9.2 ± 2.0	13.2 ± 1.7	17.6 ± 2.5 *	17.8 ± 2.6 *
AST (U/L)	144 ± 3	214 ± 24	168 ± 5	204 ± 30
Bilirubin (mg/dL)	0.18 ± 0.02	0.17± 0.008	0.17 ± 0.02	0.16 ± 0.03

**Table 3:** Changes in plasma cytokine levels at different times of liver damage.

	1 Week	2 Weeks	4 Weeks
Cytokine	Mean ± SEM (% of Controls)	Mean ± SEM (% of Controls)	Mean ± SEM (% of Controls)
TNF-α	295 ± 80 *	348 ± 77 *	266 ± 51 **
INF-γ	123 ± 6 *	103 ± 8	111 ± 8
IL-6	120 ± 6 *	110 ± 9	98 ± 8
IL-15	148 ± 18 *	141 ± 9 ***	104 ± 7
IL-17	108 ± 13	139 ± 8 *	105 ± 9
IL-4	105 ± 4	100 ± 5	139 ± 8 ***
IL-10	108 ± 9	114 ± 8	62 ± 9 **
TGF-β	108 ± 6	94 ± 4	129 ± 10 *
CX3CL1	145 ± 14 *	146 ± 15 *	158 ± 16 **
CCL5	198 ± 32 *	110 ± 8	112 ± 8
CCL20	100 ± 12	156 ± 17 **	89 ± 13
CCL2	106 ± 13	95 ± 12	101 ± 6

Plasma content of different cytokines was analyzed at one, two and four weeks of CCl4 administration. The levels of TNF-α were analyzed by ELISA and the content of all other cytokines was analyzed by western blot. Data are the mean ± SEM of 16 rats, given as percentage of control rats. Student’s *t* test was performed. Values significantly different from control rats are indicated by asterisks, * *p* < 0.05, ** *p* < 0.01, *** *p* < 0.001.

**Table 4:** Effects of treatment with rifaximin on the changes in plasma cytokines at 2 and 4 weeks of liver damage.

	2 Weeks of Liver Damage1 Week of Rifaximin				4 Weeks of Liver Damage2 Weeks of Rifaximin		
	Mean ± SEM (% of Control Rats)				Mean ± SEM (% of Control Rats)		
	C-RIF	CCl_4_	CCl_4_-RIF		C-RIF	CCl_4_	CCl_4_-RIF
TNF-α	52 ± 22	348 ± 77 *	115 ± 83 ^a^	TNF-α	119 ± 29	266 ± 51 *	138 ± 42
IL-15	98 ± 10	147 ± 15 *	104 ± 9 ^a^	IL-4	116 ± 8	139 ± 8 **	107 ± 9 ^a^
IL-17	118 ± 13	143 ± 12 *	139 ± 8 *	IL-10	91 ± 8	62 ± 9 **	61 ± 8 **
CX3CL1	119 ± 12	146 ± 15 *	157 ± 17 *	TGF-β	135 ± 10 *	141 ± 14 *	97 ± 9 ^a^
CCL20	197 ± 22*	235 ± 31 *	97 ± 16 ^a,b^	CX3CL1	81 ± 13	158 ± 16 **	86 ± 20 ^a^

The effects of treatment with rifaximin on the changes in plasma cytokines induced by liver damage were assessed at two weeks of CCl_4_ injection and one week of treatment with rifaximin and at four weeks of CCl_4_ injection and two weeks of treatment with rifaximin The levels of TNF-α were analyzed by ELISA and the content of all other cytokines was analyzed by western blot.One-way ANOVA with Tukey’s test for multiple comparisons was performed). Values significantly different from control rats are indicated by asterisks, * *p <* 0.05, ** *p <* 0.01. Values significantly different from CCl_4_ rats by a and from C-RIF group by b. a *p <* 0.05, b *p <* 0.05.

## Data Availability

All data generated or analyzed during this study are included in this published paper.

## Supplementary Materials

The following are available online at https://www.mdpi.com/article/10.3390/biomedicines9081002/s1, Figure S1: Cytometry plots: gating strategy and representative plots.
