# Supplementary material for: Rifaximin Prevents T-Lymphocytes and Macrophages Infiltration in Cerebellum and Restores Motor Incoordination in Rats with Mild Liver Damage
Source: Biomedicines. 2021 Aug 12;9(8):1002. doi: 10.3390/biomedicines9081002 (PMC8393984; doi:10.3390/biomedicines9081002)

Gating Strategy

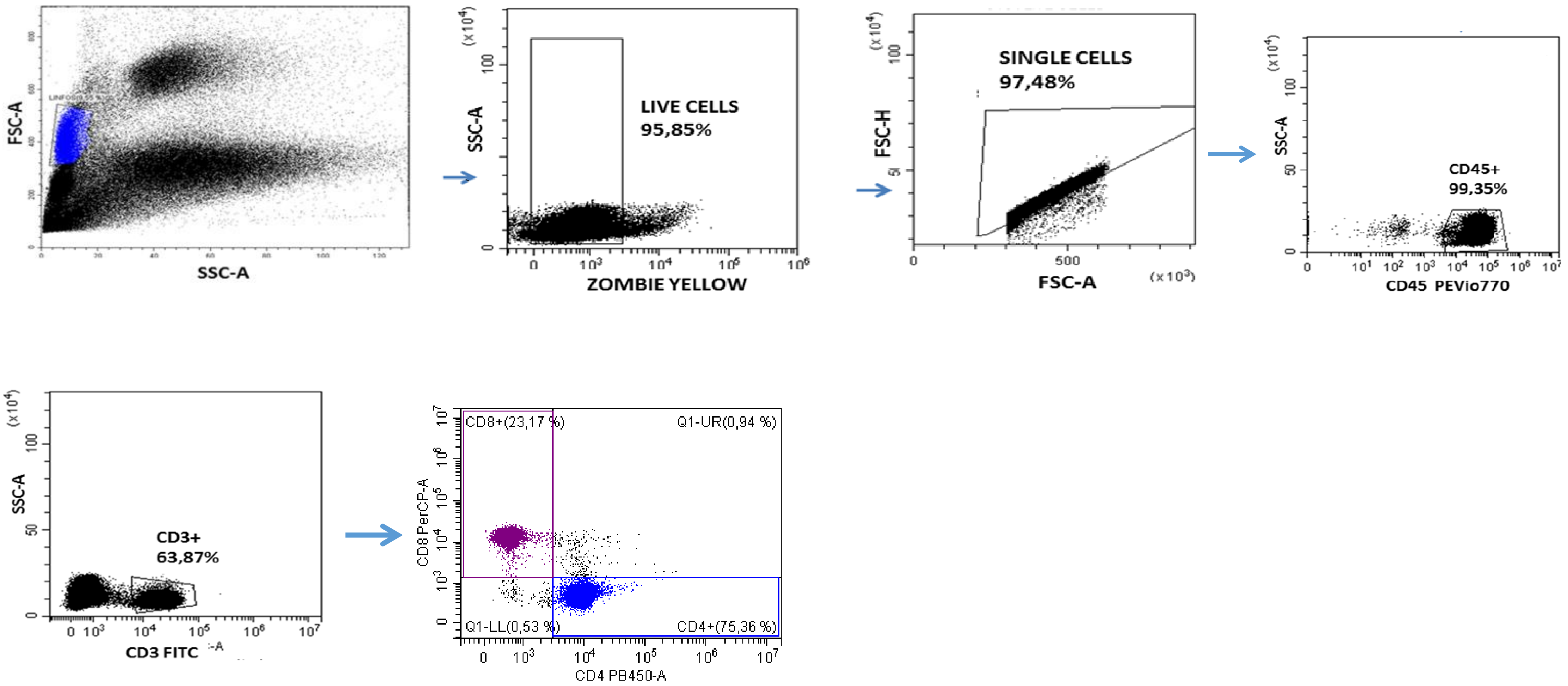

Gating Strategy

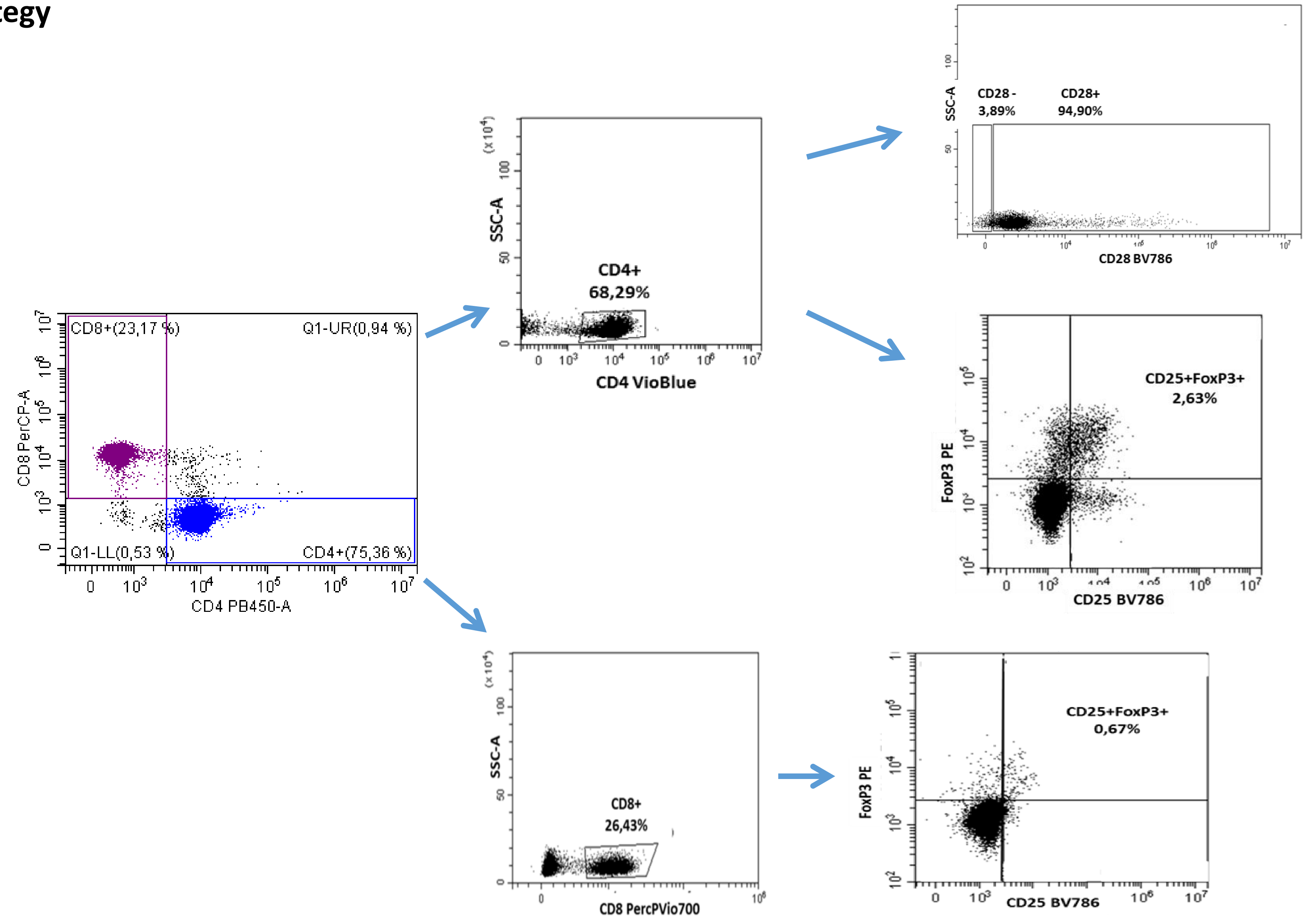

Figure 3A

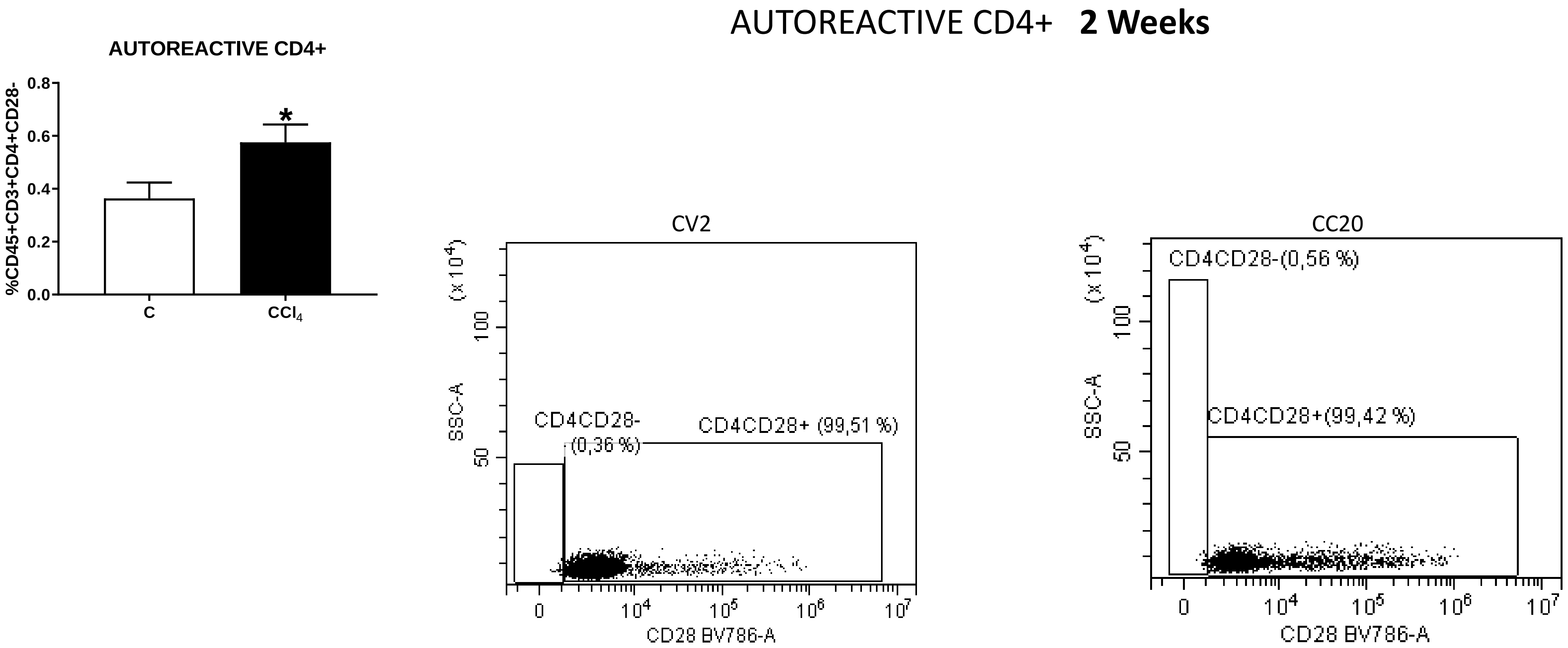
AUTOREACTIVE CD4+ 2 Weeks
AUTOREACTIVE CD4+
%CD45+CD3+CD4+CD28-
0.8
0.6
0.4
0.2
0.0
*
C
CCl4
CV2
(x 10^4)
100
50
SSC-A
CD4CD28-
(0,36 %)
CD4CD28+ (99,51 %)
0
10^4
10^5
10^6
10^7
CD28 BV786-A
CC20
CD4CD28-(0,56 %)
CD4CD28+(99,42 %)

Figure 3D

AUTOREACTIVE CD4+ **4 Weeks**

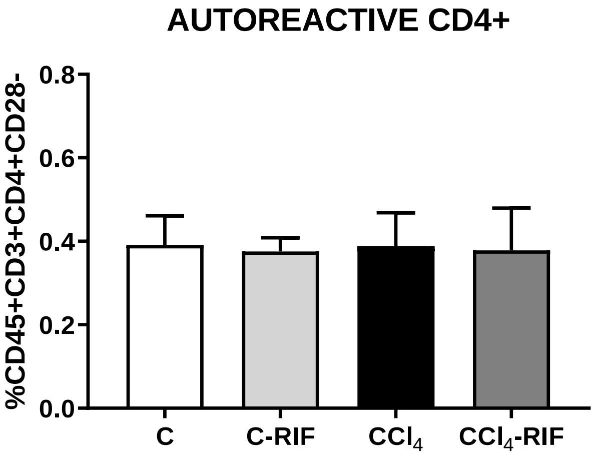

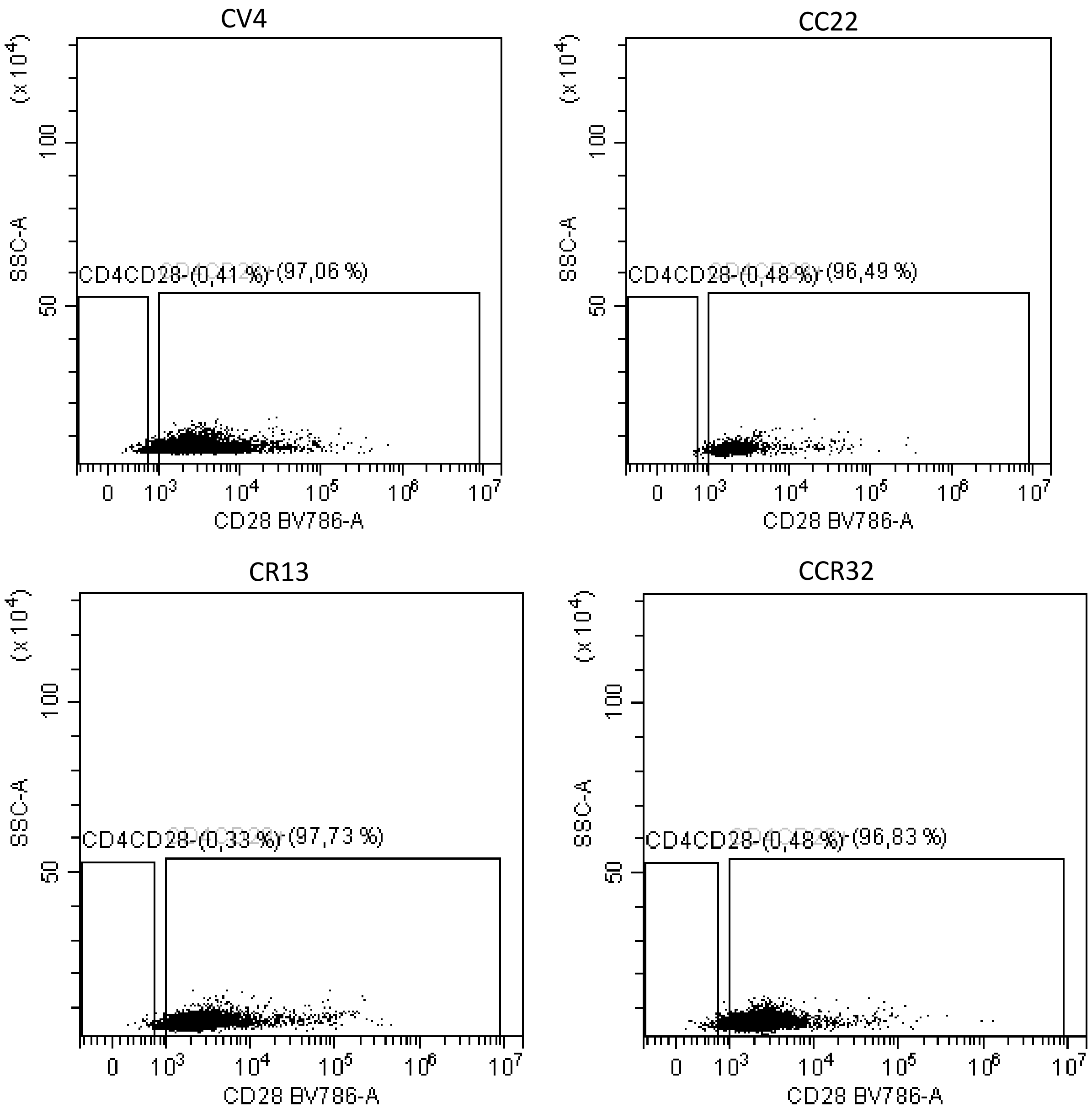

Figure 3B

REGULATORY T CELLS CD8 + **2 Weeks**

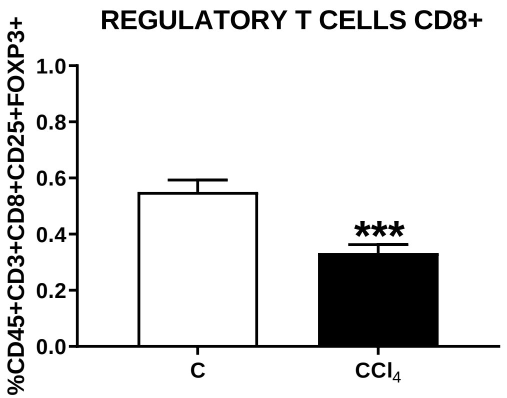

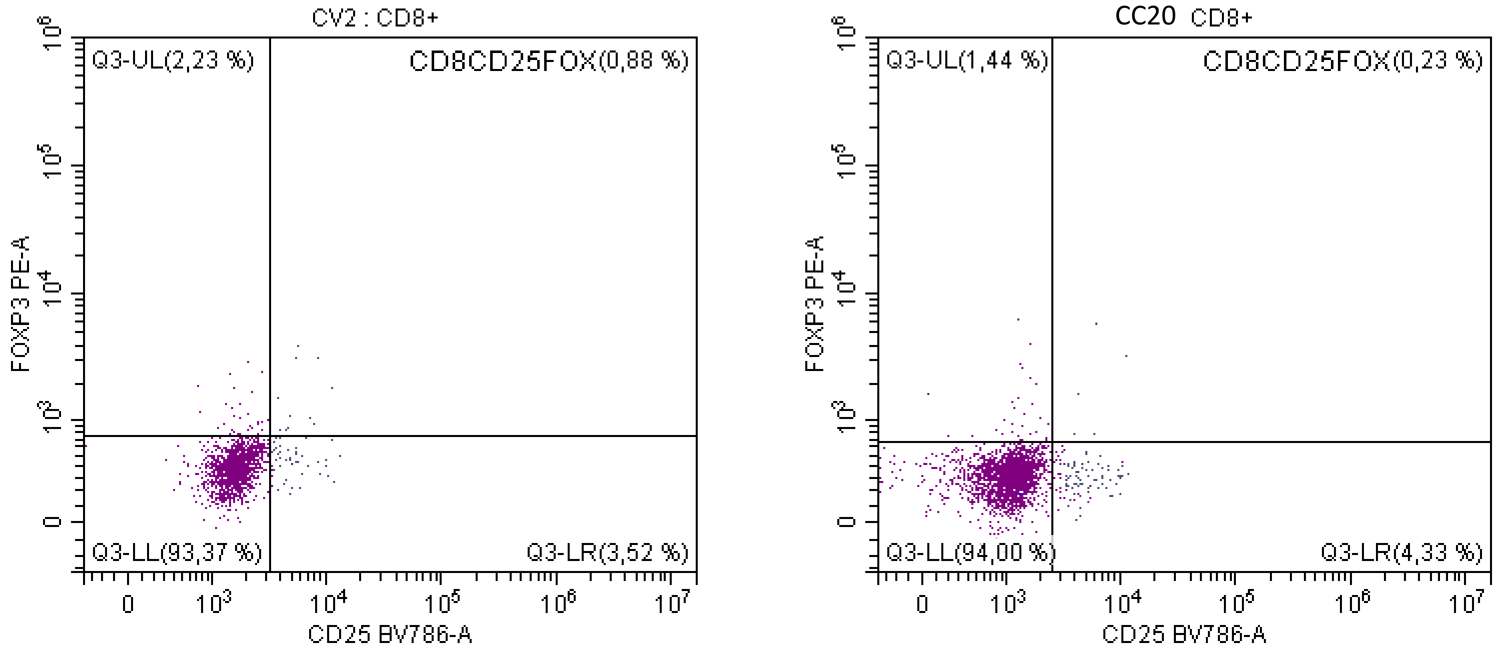

Figure 3C

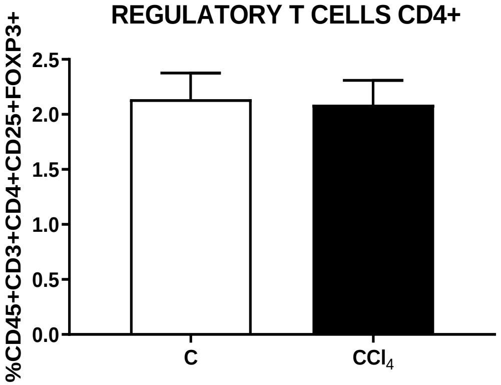

REGULATORY T CELLS CD4 + **2 Weeks**

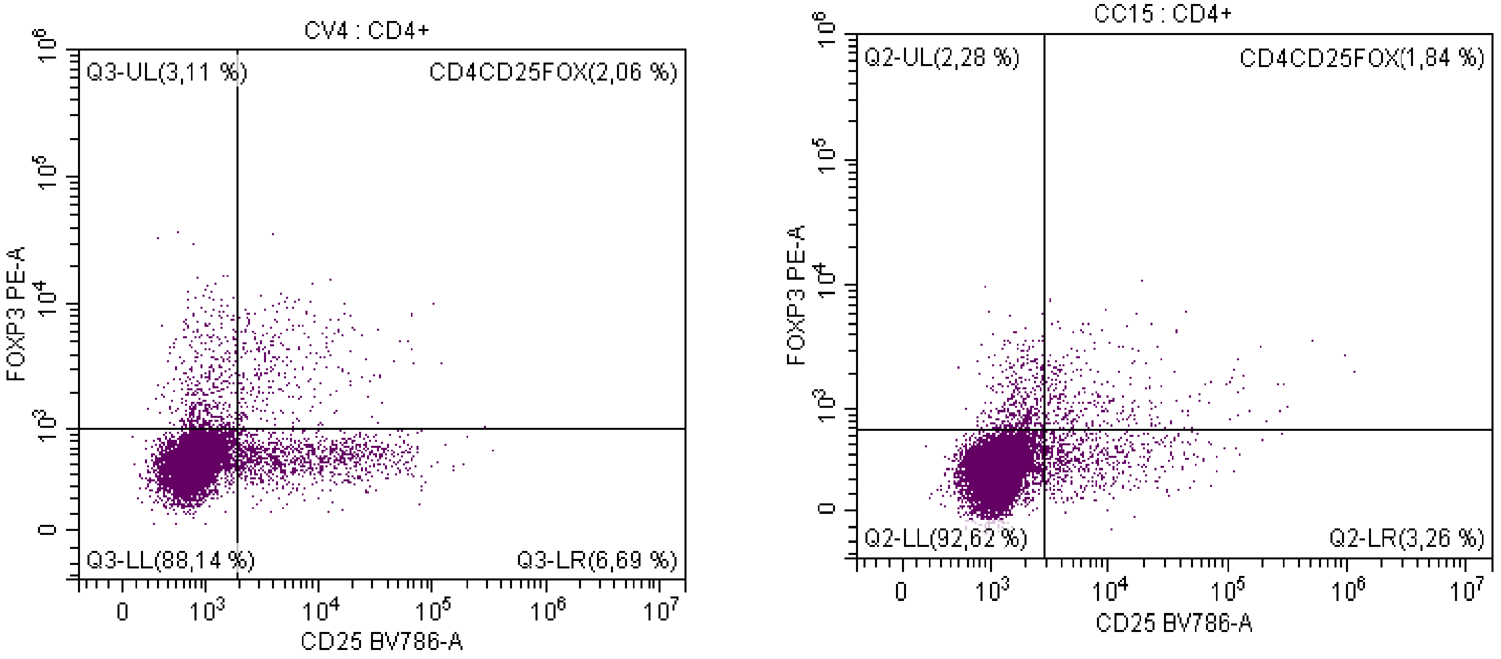

REGULATORY T CELLS CD8 + **4 Weeks**

Figure 3E

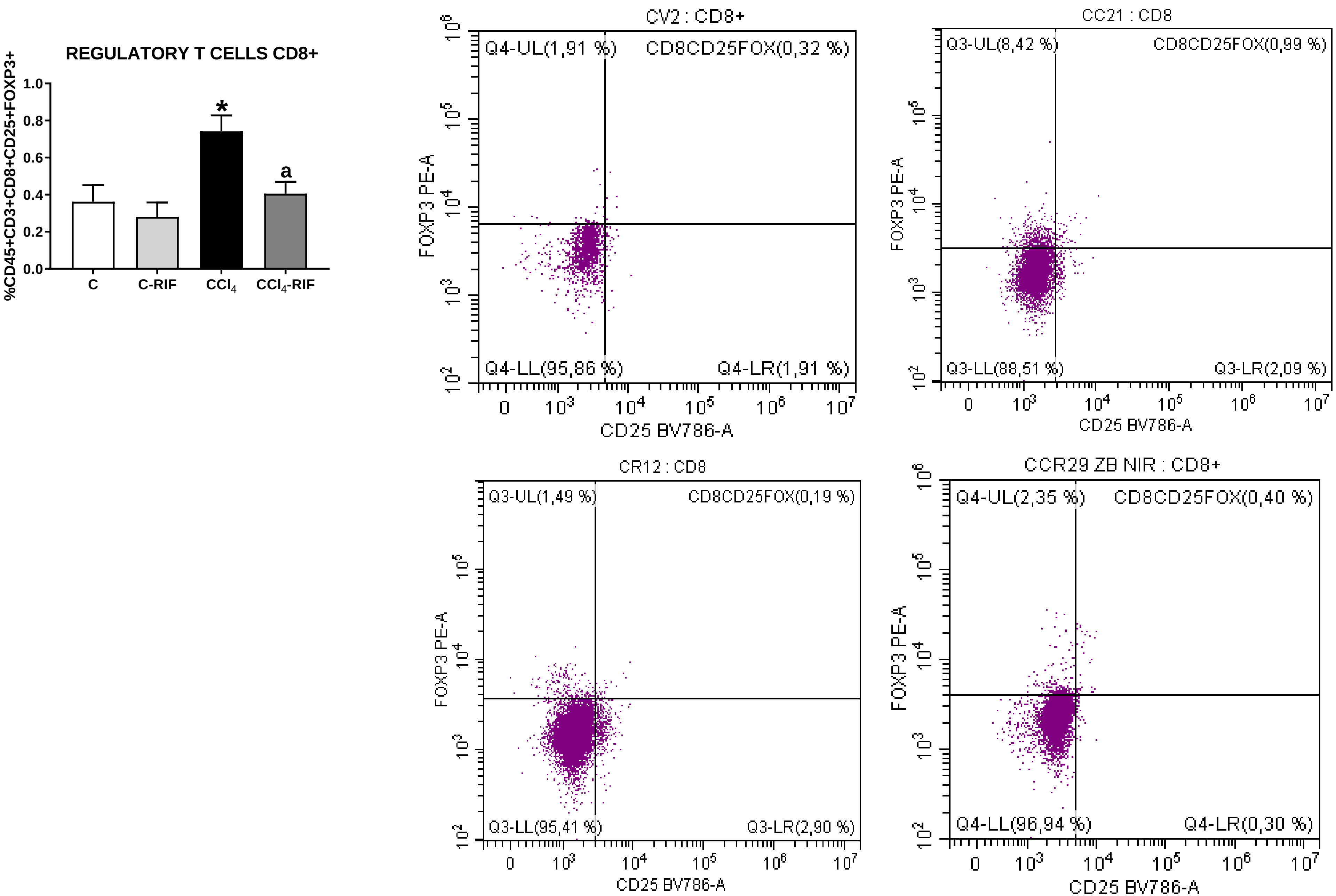

Figure 3F

REGULATORY T CELLS CD4 + **4 Weeks**

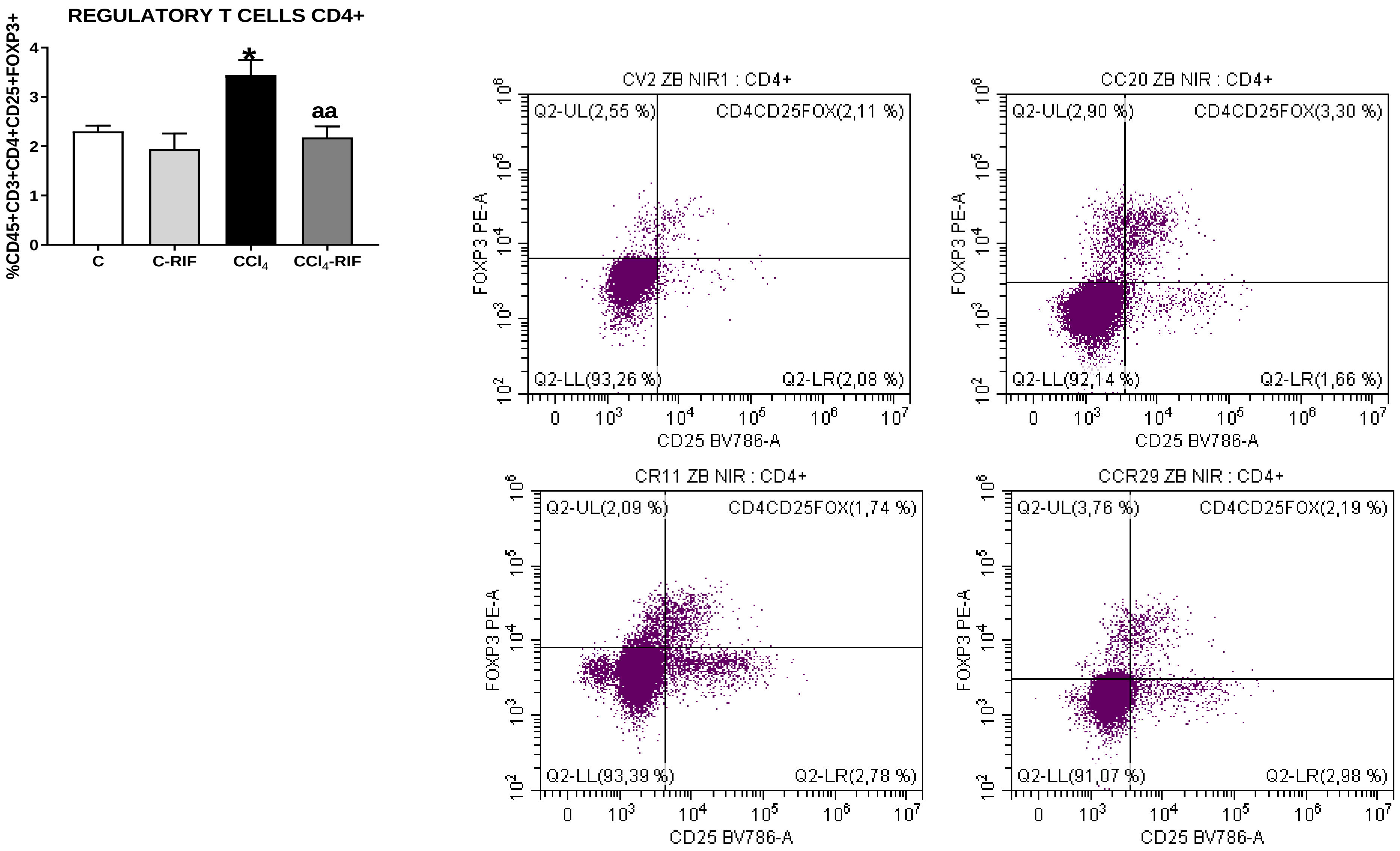

Supplement: Supplementary file 1 [file biomedicines-09-01002-s001.zip › Supplementary Figure 1. Cytometry plots.pdf]
