# Supplementary figures and images for: Rifaximin Prevents T-Lymphocytes and Macrophages Infiltration in Cerebellum and Restores Motor Incoordination in Rats with Mild Liver Damage
Source: Biomedicines. 2021 Aug 12;9(8):1002. doi: 10.3390/biomedicines9081002 (PMC8393984; doi:10.3390/biomedicines9081002)

TNF- $\alpha$

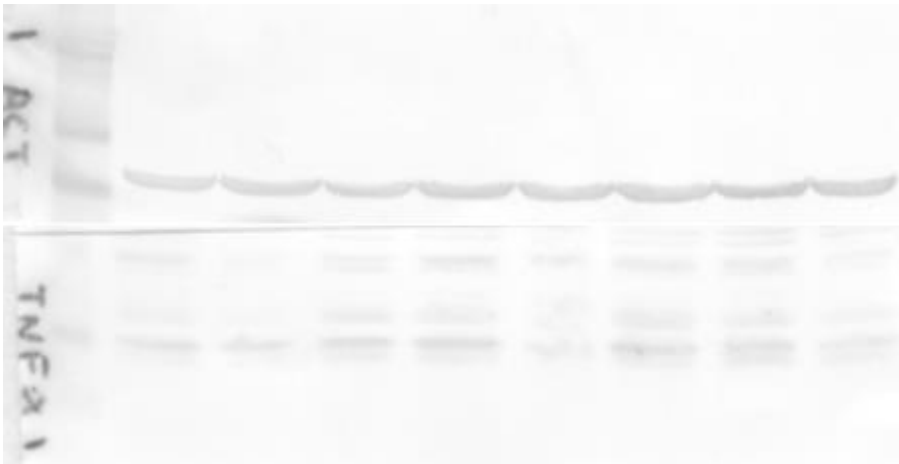

IL-6

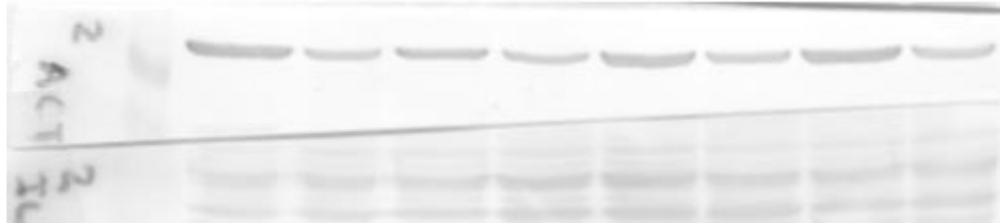

## IL-18

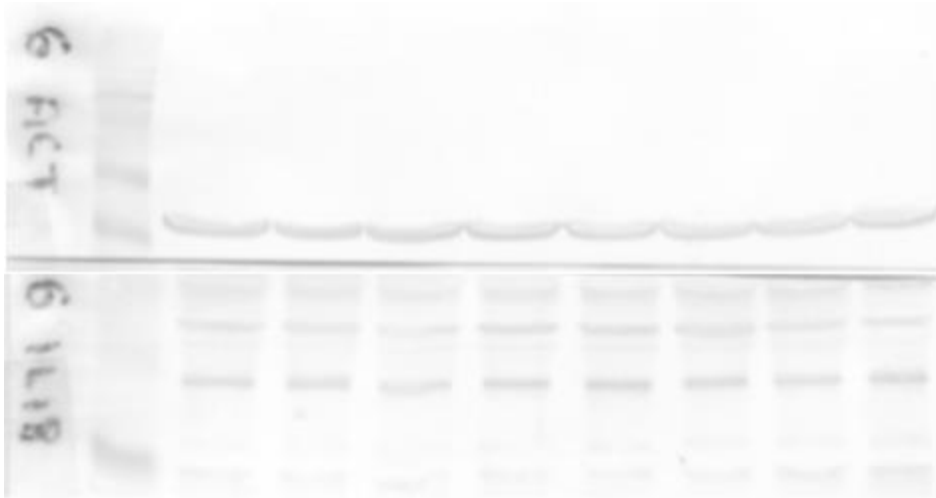

## IL-17

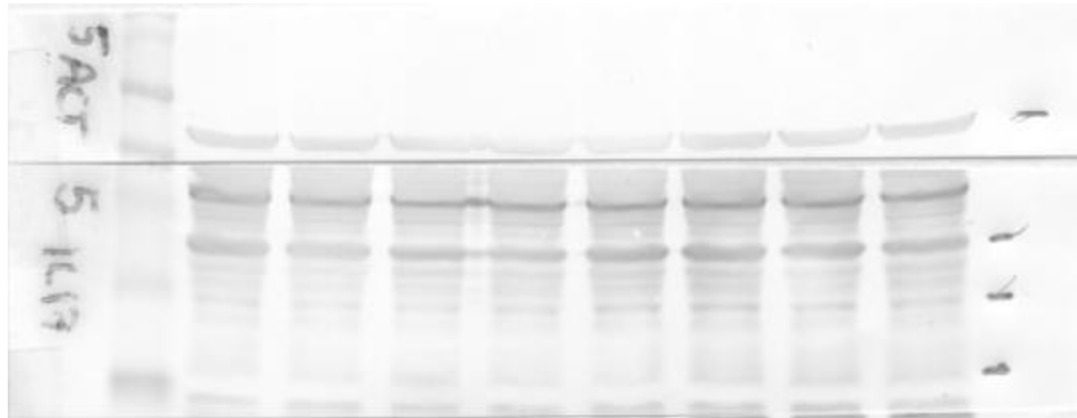

## IL-15

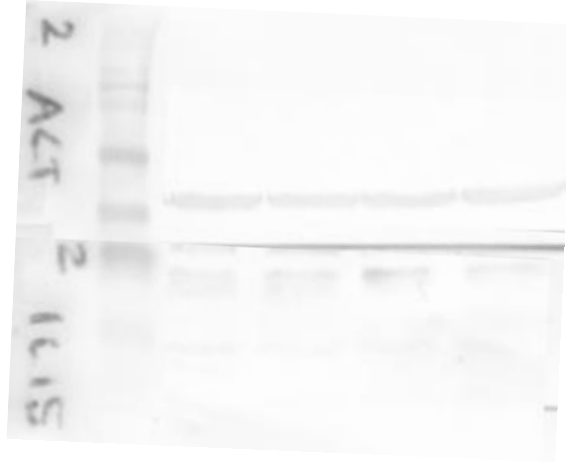

TGF-β

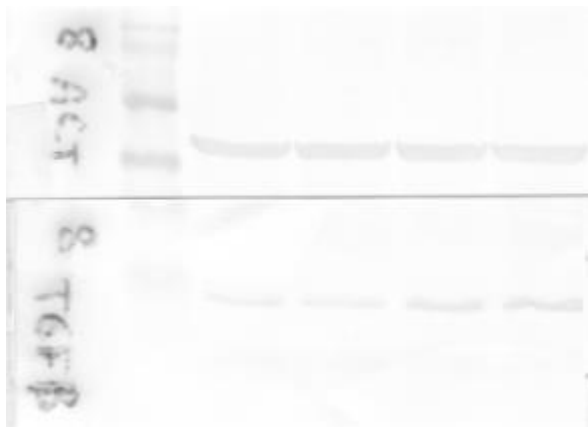

## IL-10

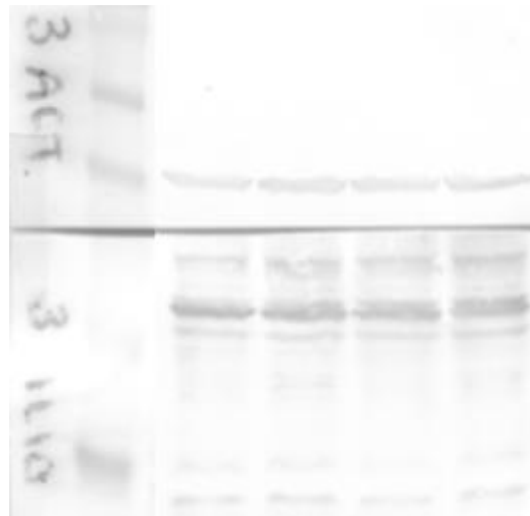

IL-4

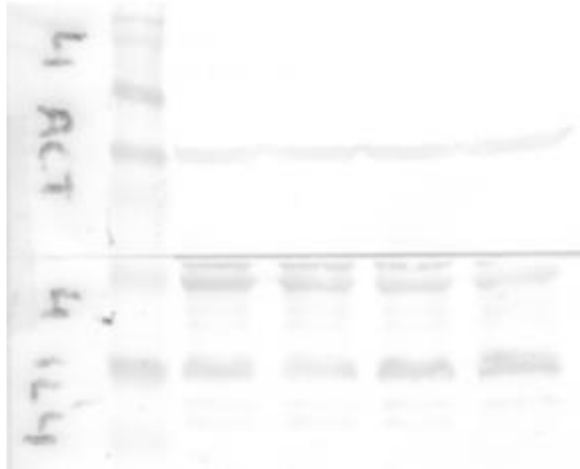

Supplement: Supplementary file 1 [file biomedicines-09-01002-s001.zip › ORIGINAL BLOT IMAGES LIVER CYTOKINES TABLE 1A R1.pdf]
